# Novel Tryptanthrin Derivatives with Selectivity as *c*–Jun N–Terminal Kinase (JNK) 3 Inhibitors

**DOI:** 10.3390/molecules28124806

**Published:** 2023-06-16

**Authors:** Igor A. Schepetkin, Oleksander S. Karpenko, Anastasia R. Kovrizhina, Liliya N. Kirpotina, Andrei I. Khlebnikov, Stepan I. Chekal, Alevtyna V. Radudik, Maryna O. Shybinska, Mark T. Quinn

**Affiliations:** 1 Department of Microbiology and Cell Biology, Montana State University, Bozeman, MT 59717, USA; igor@montana.edu (I.A.S.); liliya@montana.edu (L.N.K.); 2 O.V. Bogatsky Physico-Chemical Institute, National Academy of Sciences of Ukraine, 65080 Odesa, Ukraine; alex_chem_2@ukr.net (O.S.K.); alevtina200154@gmail.com (A.V.R.); marina_shibinskaya@ukr.net (M.O.S.); 3 Kizhner Research Center, Tomsk Polytechnic University, Tomsk 634050, Russia; anaskowry@gmail.com (A.R.K.); aikhl@chem.org.ru (A.I.K.); 4 Department of Organic Chemistry, Faculty of Chemistry and Pharmacy, Odesa I.I. Mechnikov National University, 65082 Odesa, Ukraine; stefanchekal99@gmail.com

**Keywords:** anti-inflammatory, *c*-Jun N-terminal kinase, molecular docking, nuclear factor-κB, oxime, selective kinase inhibitor, tryptanthrin

## Abstract

The *c*-Jun N-terminal kinase (JNK) family includes three proteins (JNK1-3) that regulate many physiological processes, including cell proliferation and differentiation, cell survival, and inflammation. Because of emerging data suggesting that JNK3 may play an important role in neurodegenerative diseases, such as Alzheimer’s disease (AD) and Parkinson’s disease, as well as cancer pathogenesis, we sought to identify JNK inhibitors with increased selectivity for JNK3. A panel of 26 novel tryptanthrin-6-oxime analogs was synthesized and evaluated for JNK1-3 binding (K_d_) and inhibition of cellular inflammatory responses. Compounds **4d** (8-methoxyindolo[2,1-*b*]quinazolin-6,12-dione oxime) and **4e** (8-phenylindolo[2,1-*b*]quinazolin-6,12-dione oxime) had high selectivity for JNK3 versus JNK1 and JNK2 and inhibited lipopolysaccharide (LPS)-induced nuclear factor-κB/activating protein 1 (NF-κB/AP-1) transcriptional activity in THP-1Blue cells and interleukin-6 (IL-6) production by MonoMac-6 monocytic cells in the low micromolar range. Likewise, compounds **4d**, **4e**, and pan-JNK inhibitor **4h** (9-methylindolo[2,1-*b*]quinazolin-6,12-dione oxime) decreased LPS-induced *c*-Jun phosphorylation in MonoMac-6 cells, directly confirming JNK inhibition. Molecular modeling suggested modes of binding interaction of these compounds in the JNK3 catalytic site that were in agreement with the experimental data on JNK3 binding. Our results demonstrate the potential for developing anti-inflammatory drugs based on these nitrogen-containing heterocyclic systems with selectivity for JNK3.

## 1. Introduction

JNKs are members of the mitogen-activated protein kinase (MAPK) family that regulates many physiological processes [1,2]. The JNK pathway is a highly complex pathway within the MAPK signaling network [3]. Activation of JNKs can be induced by a number of proteins, such as other MAPKs and G protein-coupled receptors (GPCRs), which transmit information into the JNK signaling pathway [4]. Despite their name, activated JNKs do not only phosphorylate *c*-Jun, and close to 100 JNK substrates are known [5]. However, *c*-Jun is a major substrate for JNKs, and its phosphorylation is closely tied to activator protein 1 (AP-1) activation. Previous studies have shown that JNKs play an important role in the regulation of signaling pathways involved in inflammation, apoptosis, and necrosis [6,7,8,9]. Indeed, JNK signaling is involved in a wide range of diseases, including multiple sclerosis, rheumatoid arthritis, osteoarthritis, insulin resistance, inflammatory bowel diseases, cancer, stroke, renal ischemia, essential hypertension, Alzheimer’s disease, and Parkinson’s disease [10,11,12,13,14,15,16,17,18,19,20,21]. Therefore, JNKs have become important therapeutic targets and many groups have sought to develop inhibitors for these targets [22,23,24]. Nevertheless, further development of novel JNK inhibitors with clinical value is essential.

The human genome contains three closely related JNK genes (JNK1, JNK2, and JNK3), with each gene encoding multiple isoforms [25]. While JNK1 and JNK2 are expressed in a variety of tissues, JNK3 expression has been reported to be limited mainly to neuronal tissue and, to a lesser extent, heart and testes [26,27]. Thus, most early studies on JNK3 function have focused primarily on its role in brain and neural tissues. However, more recent studies suggest that JNK3 is involved in a wider range of tissues and pathologies. For example, JNK3 has been reported to be expressed in human and mouse pancreatic *β*-cells [28], human CD14^+^ monocytes [29], normal human epithelial cells [30], normal human MRC-5 fibroblasts, and human thyroid cells and tissue [31]. While the function of JNK3 in these tissues is still being defined, several reports have suggested important regulatory functions. JNK3 has been reported to play an important role in the circulatory system, including in angiogenesis [32]. Likewise, it has been reported that β-arrestin 2 can regulate macrophage function via JNK3 [33]. Additional studies on the patterns of JNK3 expression in various tissues may find that JNK3 has both compensatory and opposing functions that affect the ability of JNK1 and JNK2 to carry out their characteristic activities [34].

Recent research has indicated that JNK3 may play an important role in cancer. For example, JNK3 was found to be expressed in human ovarian cancer SKOV3/DDP cells [35], human hepatocellular carcinoma cells [36], human small-cell lung cancer cells [37], human esophageal squamous cell carcinoma cells [38], and HeLa cells [39]. Notably, Gorogh et al. [40] reported that JNK3 was capable of conveying chemotherapy resistance and survival in HNSCC (head and neck squamous cell carcinoma) cells. Likewise, analysis of prostate cancer clinical samples indicated that high intratumor JNK3 levels correlated with a worse prognosis [41]. In human breast cancer samples and tumor cell lines, JNK3, but not the other JNK isoforms, responded to amyloid precursor protein (APP) signaling, and subsequent JNK3 phosphorylation facilitated epithelial–mesenchymal transition of breast cancer cells [42]. Finally, high basal levels of JNK3 expression were present in oral keratinocyte cell lines, and invasion by these cells was found to be primarily JNK3-dependent [43].

**Figure 1:**
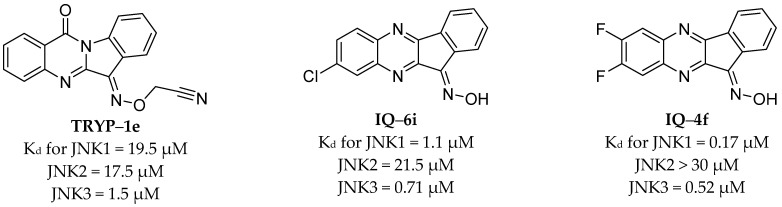
Chemical structures of JNK inhibitors based on tryptanthrin and 11*H*-indeno[1,2-*b*]quinoxalin-11-one scaffolds with a relative high JNK isoform selectivity [49,50,51].

More than 100 JNK inhibitors have been identified, with some even evaluated in clinical trials, yet fewer than ten JNK3-selective inhibitors have been reported [44,45,46,47]. Tryptanthrin (TRYP) (indolo[2,1-*b*]quinazolin-6,12-dione) is an alkaloid that can be isolated from higher plants and several marine organisms (for a review, see [48]). We previously found that tryptanthrin-6-oxime (TRYP-Ox) derivatives and their analogs with an 11*H*-indeno[1,2-*b*]quinoxalin-11-one scaffold were effective JNK inhibitors [49,50,51,52] (see Figure 1). Notably, the TRYP-Ox derivative **TRYP**–**1e** had ~10-fold greater selectivity for JNK3 binding versus JNK1/JNK2 [49] (Figure 1). In the present studies, we synthesized a new series of twenty-six TRYP-Ox derivatives in an effort to identify novel and isoform-selective JNK inhibitors. Specifically, we evaluated the effects of substituents in the tetracyclic scaffold of TRYP-Ox on enzymatic binding, cell-mediated inflammatory responses, and *c*-Jun phosphorylation.

## 2. Results and Discussion

### 2.1. Synthesis of Tryptanthrin Derivatives

Our prior analysis of indenoquinoxaline and tryptanthrin derivatives demonstrated that an oxime group was essential for JNK inhibition, and compounds lacking the oxime had little or no effect on JNK activity [49,50,53,54]. Therefore, we examined the effect of adding different substituents to the tetracyclic scaffold of tryptanthrin while maintaining the oxime group. JNK binding and inhibitory activity of these TRYP-Ox derivatives were evaluated.

**Scheme 1:**
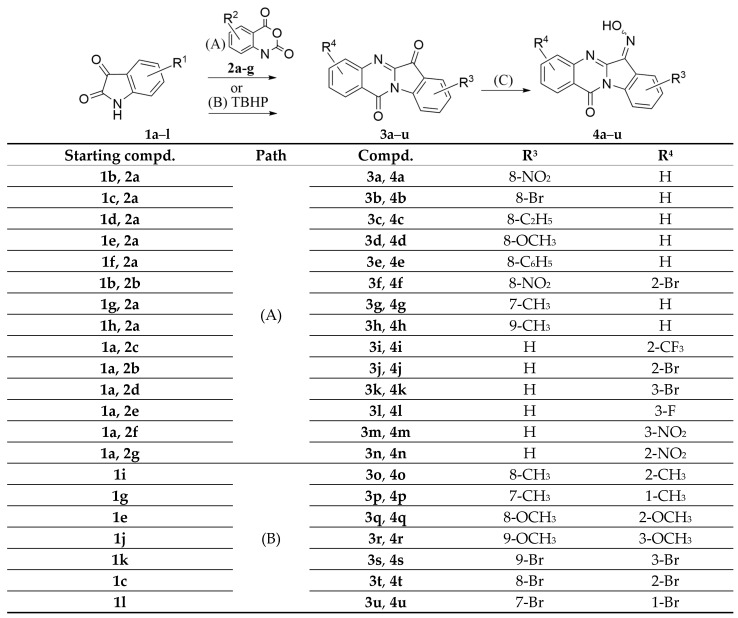
Reagents and conditions: (**A**) isatoic anhydride, triethylamine, toluene, reflux, 1–2 h, 35–60%; (**B**) K_3_PO_4_, *tert*-butyl hydroperoxide (TBHP), dimethyl sulfoxide (DMSO), r.t., 20 h, 20–95%; (**C**) pyridine, NH_2_OH·HCl, reflux, 4 h, 85–95%.

For compound synthesis, substituted isatins **1a**–**l** and isatoic anhydrides **2a**–**g** (see Table 1) were refluxed in boiling toluene (Scheme 1, path A) to obtain the corresponding ketones **3a**–**n**. Poor yields were observed when electron-withdrawing substituents were present in position 8 (NO_2_, Br), and only traces of target compounds were obtained when starting with 7-substituted isatins.

The procedure shown in path B, described previously in [55], was used for synthesis of pseudo-symmetric ketones **3o**–**u**. Corresponding target compounds were obtained in low yields, although low yields were compensated for by simplicity of the procedure. Using 7-methyl, 7-methoxy, or 5-nitro substituted isatins for synthesis via path A gave just traces of the corresponding target products.

Oximes **4a**–**u** were synthesized from ketones **3a**–**u** by refluxing in pyridine and then treating with excess of hydroxylamine hydrochloride. The general yields of this procedure were high. Minor impurities and traces of starting compounds were removed by washing with water or boiling toluene, and with recrystallization from 1,4-dioxane.

We also considered the possibility of obtaining active tryptanthrin derivatives by modifying the oxime fragment using *O*-substitution [50]. One of the methods for obtaining aryl (Ar) derivatives with an isonitroso group (Ar=N-OR) is the reaction of tryptanthrin with *O*-substituted hydroxylamine hydrochlorides. However, this reaction is limited in the choice of reagents in so far as *O*-substituted hydroxylamines mainly have nonionic groups (e.g., alkyl and halogen derivatives) [56]. It is worth noting that the dependence of reaction mechanisms on the substrates used and reaction conditions has not been previously described in the literature.

**Scheme 2:**
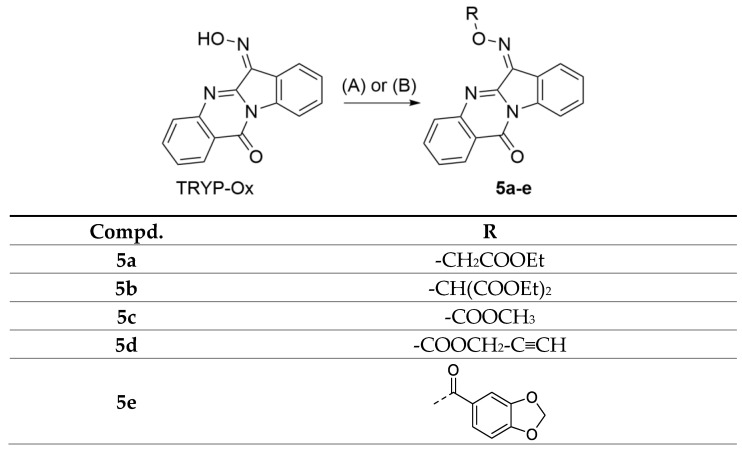
Reagents and conditions: (**A**) alkylating agent, KOH, DMSO, r.t. (compounds **5a**–**b**); (**B**) acylating agent, pyridine, 0 °C (compounds **5c**–**e**).

*O*-substitution reactions were performed to obtain compounds **5a**–**e** in a superbasic medium (KOH/DMSO), which revealed dependence of the reactivity of oximes on these conditions. We considered a number of acylating and alkylating reagents, such as methyl chloroformate, propargyl chloroformate, piperonyl chloride, ethyl ester of monochloroacetic acid, and diethyl ester of bromomalonic acid. Synthesis of oxime analogues **5c**–**e** by acyl substitution in the KOH/DMSO system (Scheme 2, path A) failed. Presumably, this was due to the fact that DMSO, being an aprotic solvent, easily solvates potassium cations, while anions are only slightly solvated, which in turn leads to superbasic properties of the medium [57] and a decrease in stability of the intermediate in the acyl substitution reaction. Another reason why it was not possible to carry out reactions with chloroformates in the KOH/DMSO system may be the basic hydrolysis of formates to salts of organic acids and reaction products to the starting oximes. However, the acyl substitution occurred under milder conditions, such as in pyridine, which simultaneously acts as a solvent and a base (Scheme 2, path B).

Nucleophilic substitution in the synthesis of compounds **5a**–**b** was facilitated in the superbasic medium, probably due to an easier formation of the oximate nucleophile. Completion of the reactions shown in Scheme 2 was monitored with TLC (eluent: chloroform).

### 2.2. Binding Affinity of the Compounds for JNK1-3

All compounds were evaluated for their ability to bind to JNK1-3 using the KINOMEscan platform [58]. This method was validated for the structurally related 11*H*-indeno[1,2-*b*]quinoxalin-11-one JNK inhibitor IQ-1S by an independent fluorescence polarization-based competition binding assay [59].

One compound (**4h**) had high affinity for JNK, with K_d_ values in the submicromolar range for all three JNK isoforms, whereas seven compounds (**4a**, **4d**, **4e**, **4i**, **4k**, **4m**, and **4n**) were selective for JNK3 and did not bind to either JNK1 or JNK2 (Table 2). Note that four compounds with relatively high selectivity (selectivity index > 7) had a single substituent at R_2_ (i.e., NO_2_, Br, OMe, and phenyl in compounds **4a**, **4b**, **4d**, and **4e**, respectively). Moving the methyl group in compound **4g**, which had relatively high selectivity for JNK3, from R_1_ to R_3_ led to compound **4h**, which had low selectivity but high binding activity for all JNK isoforms. Moving NO_2_ from R_2_ (compound **4a**) to R_5_ (compound **4n**) or R_6_ (compound **4m**) decreased binding affinity for JNK3 and reduced selectivity. Introduction of an additional OMe group in compound **4d** or an additional Me in compound **4g** led to inactive compounds **4q** and **4p**, respectively. Similarly, introduction of additional Br in compounds **4a**, **4b**, and **4k**, led to inactive compounds **4f**, **4t**, and **4s**, respectively, which is probably due to the bulkiness of the molecule and decreased complementarity to the binding site.

Among the *O*-substituted TRYP-Ox derivatives, compounds **5a** and **5b** were inactive (**5a**) or had low binding activity (**5b**) for all JNK isoforms, whereas the other three compounds **5c**–**e** had relatively high affinity, with K_d_ values in the submicromolar range for all three JNK isoforms, indicating low JNK isoform selectivity (Table 2).

### 2.3. Activity of Compounds in Monocytic Cells

Prior to evaluation of the compounds in cell-based assays, we measured their cytotoxicity in human monocytic THP-1Blue and MonoMac-6 cells during a 24 h incubation. Seven compounds (**4a**, **4f**, **4i**, **4j**, and **4r**–**t**) were cytotoxic in both cell lines, with IC_50_ values ranging from 0.17 to 49.1 μM (Table 3). Compounds **4k** and **4n** exhibited cytotoxicity in THP-1Blue cells, and compounds **4c** and **5e** were cytotoxic for MonoMac-6 cells. Thus, these compounds were all excluded from subsequent testing in these cell lines.

**Figure 2:**
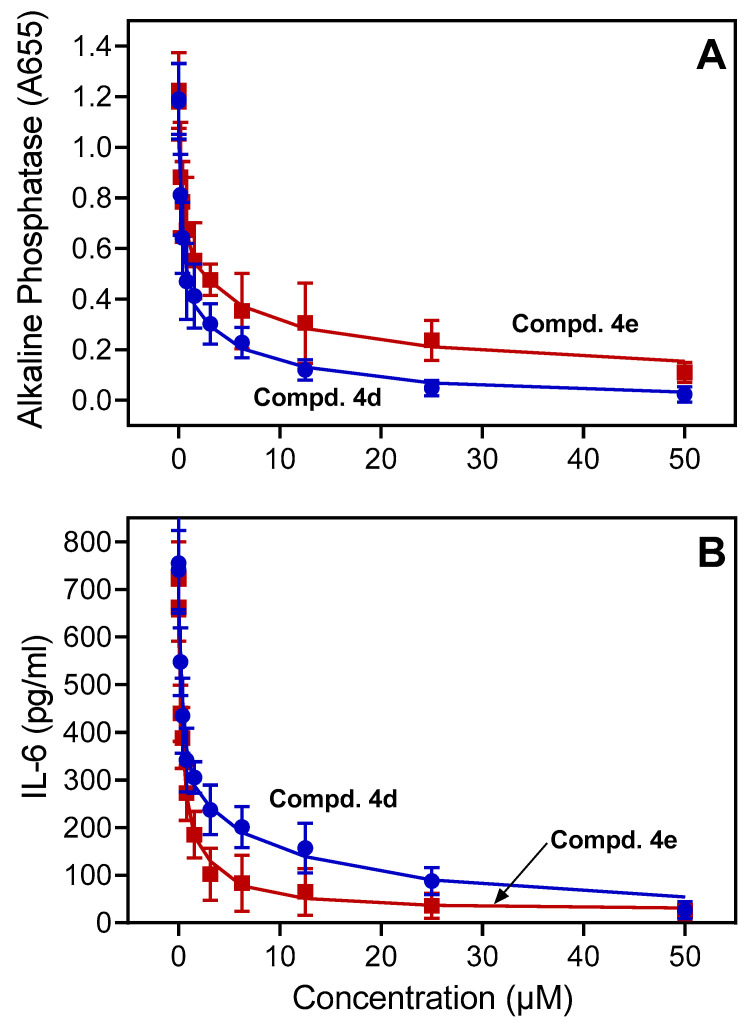
Effect of compounds **4d** and **4e** on NF-κB/AP-1 activity and IL-6 production. Panel (**A**) THP-1Blue cells were pretreated with the indicated compounds or DMSO for 30 min, followed by addition of 250 ng/mL LPS or buffer for 24 h. NF-kB/AP-1 activity was monitored by measuring secreted alkaline phosphatase activity spectrophotometrically in the cell supernatants (absorbance at 655 nm). Panel (**B**) MonoMac-6 cells were pretreated with the indicated compounds or DMSO for 30 min, followed by addition of 250 ng/mL LPS or buffer for 24 h. Production of IL-6 in the supernatants was evaluated with ELISA. The data in each panel are presented as the mean ± S.D. of triplicate samples from one experiment that is representative of three independent experiments.

The JNK pathway can be activated through Toll-like receptor 4 (TLR4), leading to the activation of transcription factors NF-κB and AP-1 [60,61]. Thus, to assess the anti-inflammatory activity of our derivatives, the remaining non-cytotoxic compounds were evaluated for their ability to inhibit lipopolysaccharide (LPS)-induced NF-κB/AP-1 reporter activity and IL-6 production in THP-1Blue and MonoMac-6 cells, respectively. Compounds **4c**–**e**, which contain ethyl, methoxy, and phenyl groups at R_2_, and compound **4h**, which has a methyl group at R_3_, were the most potent inhibitors of NF-κB/AP-1 reporter activity in THP-1Blue cells, with IC_50_ values < 2 μM (Table 3). As an example, the dose-dependent inhibition of LPS-induced NF-κB/AP-1 reporter activity by compounds **4d** and **4e** is shown in Figure 2A.

Six of the compounds (**4d**, **4e**, **4h**, **4k**, **4l**, and **4o**) were also potent inhibitors of IL-6 production in MonoMac-6 cells, with IC_50_ values < 1 μM. As an example, the dose-dependent inhibition of LPS-induced IL-6 production by compounds **4d** and **4e** is shown in Figure 2B. Note that **4d** and **4e** were highly specific for JNK3, whereas compound **4h** was a pan-JNK inhibitor. Thus, these three compounds were selected for subsequent analysis in MonoMac-6 cells for their ability to inhibit LPS-induced *c*-Jun phosphorylation.

**Figure 3:**
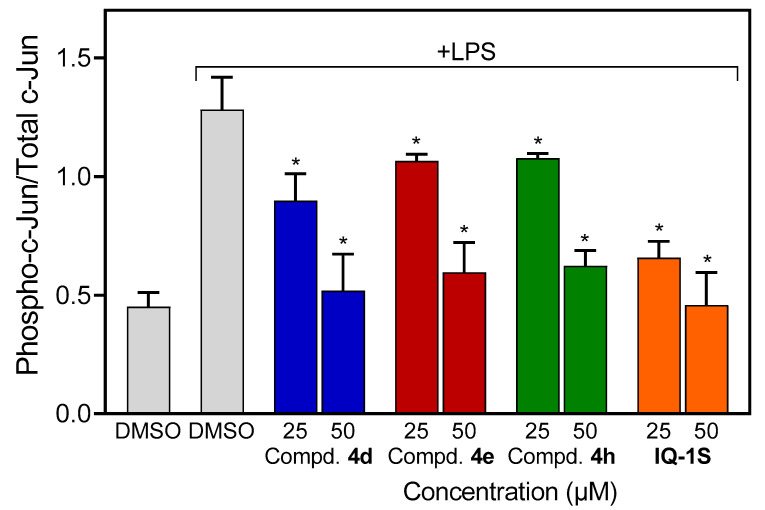
Effect of the compounds **4d**, **4e**, **4h**, and IQ-1S on LPS-induced *c*-Jun phosphorylation. Human MonoMac-6 monocytic cells were pretreated with the indicated concentrations of compounds or 0.5% DMSO for 30 min, followed by treatment with LPS (500 ng/mL) or control buffer for another 30 min. The cells were lysed, and the lysates were analyzed with ELISA for phospho-*c*-Jun (Ser63) and total *c*-Jun. The data are represented as the ratio of phospho-*c*-Jun to total *c*-Jun. The data are presented as the mean ± S.D. of triplicate samples from one experiment that is representative of three independent experiments. * Significant differences (*p* < 0.05) with LPS control.

MonoMac-6 cells were pretreated with compounds **4d**, **4e**, **4h**, or a structural analog IQ-1S with demonstrated potent JNK inhibitory activity as a positive control **[53]**. After 30 min pretreatment with the compounds, the cells were stimulated with LPS (500 ng/mL), and the level of phospho-*c*-Jun (S63) was determined by a sandwich ELISA. All of these compounds dose-dependently inhibited *c*-Jun phosphorylation (Figure 3), directly confirming JNK inhibition.

### 2.4. Molecular Modeling

To gain insight into interactions of the investigated compounds with JNK and explain some of the observations made in our structure–activity relationship analysis, we performed molecular docking of selected compounds and TRYP-Ox into the JNK3 binding site (PDB: 1PMV) using Molegro Virtual Docker 6.0 (MVD) software. Note that these compounds exist as mixtures of *Z* and *E* isomers with respect to the exocyclic C=N bond, and these geometric isomers are prone to interconversion [62,63]. MolDock docking scores for the docking poses of both isomers of these compounds are shown in Table 4.

**Figure 4:**
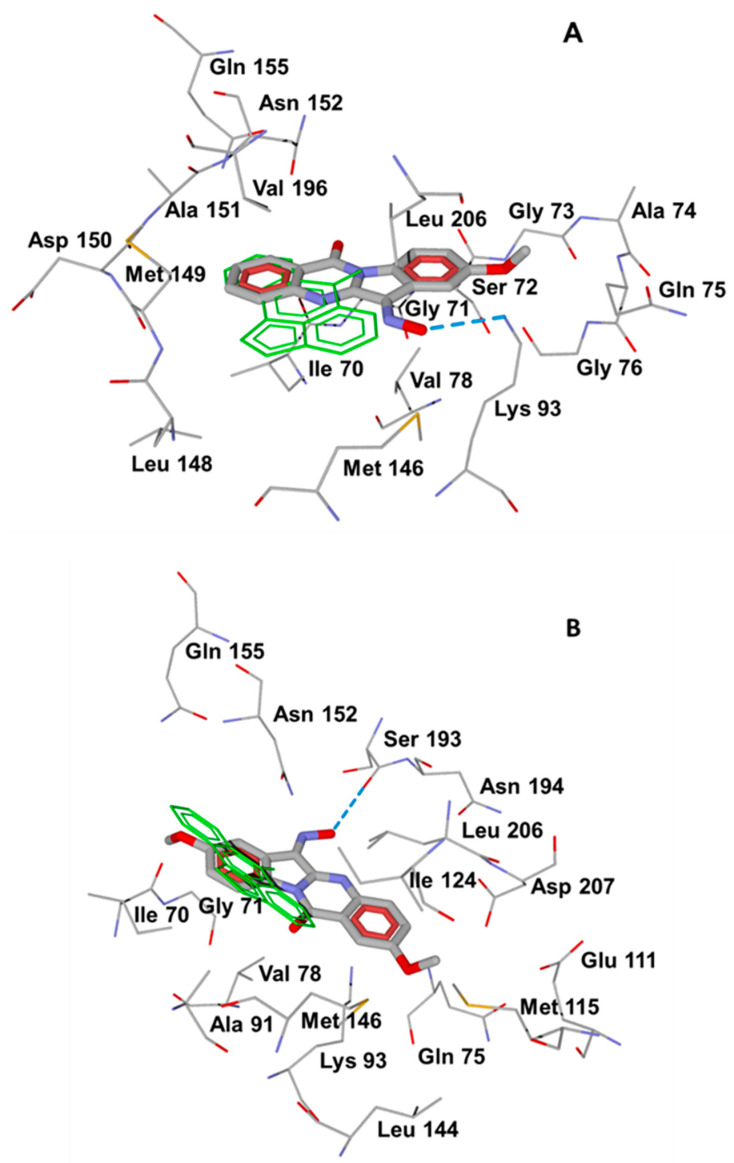
Docking poses of compounds **4d** (*E*) (Panel (**A**)) and **4q** (*Z*) (Panel (**B**)) into the JNK3 binding site (PDB: 1PMV). The co-crystallized ligand SP600125 is represented by thin green sticks. Residues within 4 Å of each pose are visible. H-bonding interactions are shown in blue dashed lines.

Compounds **4a** (*Z*), **4b** (*Z*), and **4d** (*E*) had the highest docking scores and were located within the JNK3 binding site, similar to the co-crystallized pan-JNK inhibitor SP600125. As an example, see the docking pose of the methoxy derivative **4d** (*E*) in Figure 4A, where it forms a strong H-bond to Lys93 with its oxime oxygen atom. In this binding mode, the quinazolone moiety coincides with the experimentally determined position of SP600125 in PDB structure 1PMV [64]. In contrast, the inactive compound **4q** (*Z*), which contains two methoxy groups and did not have affinity for any of the three JNK isoforms, had a different location in the JNK3 binding site. As shown in Figure 4B, molecular docking results suggested that compound **4q** (*Z*) could be H-bonded to Ser193 and deviate significantly from the plane of SP600125. This difference is likely due to steric effects introduced by adding the second methoxy group in the tryptanthrin scaffold. In the docking pose of compound **4d** (*E*), the benzene ring in the azaindene moiety is located near Met149, Asp150, and Ala151 of JNK (Figure 4A), and the additional methoxy substituent in this ring would cause a steric clash with neighboring residues. Hence, compound **4q** (*Z*) should be shifted and rotated within the binding site with respect to compound **4d** (*E*), which leads to a lower DS value (Table 4).

**Figure 5:**
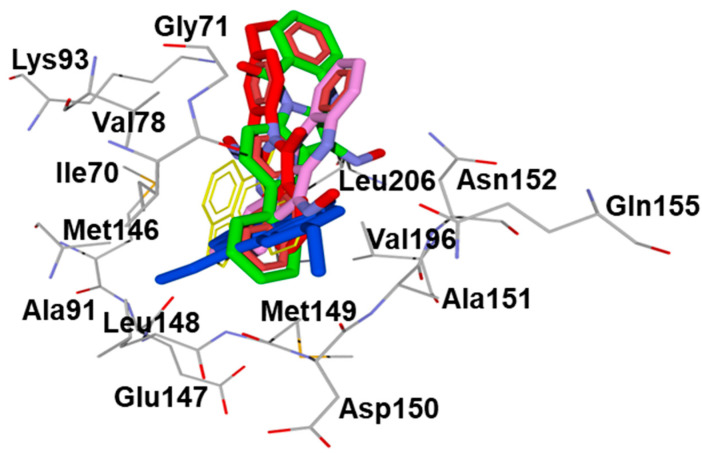
Superimposed docking poses of the most active compounds **4c** (blue), **4d** (red), **4e** (green), and **4h** (magenta). The position of the co-crystallized ligand SP600125 (PDB: 1PMV) is shown in thin yellow sticks. Residues within 4 Å from SP600125 are shown.

We also conducted molecular docking of the compounds found to be most potent in the cell-based assays (**4c**–**e**,**h**; see Table 3) to evaluate their relative positions within the JNK3 binding site. The superimposed final docking poses for these compounds are shown in Figure 5 and show that these compounds occupy approximately the same area of space in binding to the enzyme. Additionally, they overlap significantly with the position of co-crystallized SP600125 present in the PDB 1PMV structure.

To evaluate the relative energies of geometric isomers and the barrier for *Z*,*E*-isomerization, we performed DFT calculations of relative Gibbs free energies for *Z*- and *E*-isomers of compound **4d** in DMSO using the M06-2X functional, which is suitable for evaluation of thermochemical properties of organic compounds [65]. We found that the *E*-isomer had a slightly higher thermodynamic stability, with its Gibbs energy being 0.96 kcal/mol lower than that of the *Z*-isomer. Using the DFT method, we also found that *E*→*Z* isomerization occurs via in-plane inversion of the oxime nitrogen atom, similar to other molecules containing a C=N bond [66]. With the small difference in Gibbs energies indicated above, both isomers can be present in the solution [67]. However, the calculated isomerization barrier *ΔG^≠^* for oxime **4d** was 49.5 kcal/mol in DMSO, indicating that isomerization in solution should be a slow process in accordance with one of the isomers predominating in the synthesized samples (see the NMR data in Section 3). However, isomerization may occur during interaction of the investigated tryptanthrin oximes with the JNK binding site. *Z*,*E*-isomerization of these oximes in solution and on binding to the enzyme is a subject of our future studies.

## 3. Experimental Section

### 3.1. Chemistry

Starting materials for the synthesis of **4a**–**u** were obtained from Enamine (Kiev, Ukraine), while starting materials for synthesis of **5a**–**e** were obtained from Merck. Tryptanthrin (indolo[2,1-*b*]quinazolin-6,12-dione) was purchased from Combi-Blocks (San Diego, CA, USA). Reaction progress was monitored by thin-layer chromatography (TLC) with UV detection using pre-coated silica gel 60, F254 plates (Merck, Rahway, NJ, USA). Structures of the synthesized compounds were confirmed on the basis of analytical and spectral data. The melting points (mp) were determined using an electrothermal Mel-Temp capillary melting point apparatus and an SMP30 melting point apparatus, with a heating rate of 3.0 °C/min. Elemental analysis was performed with a Carlo Erba instrument. LC-HRMS analysis was performed on an Agilent chromatograph with a 6538 UHD Accurate-Mass Q-TOF detector (ESI in positive mode). EI-MS spectra were obtained from a MX-1321 instrument (T_sourse_ = 220 °C, E_ionisation_ = 70 eV). NMR spectra were recorded on Bruker spectrometers (operating frequencies: 400 and 600 MHz for ^1^H, 100 and 150 MHz for ^13^C, and 280 MHz for ^19^F). IR spectra were recorded on a Nicolet 5700 FT-IR spectrometer with KBr pellets.

TRYP-Ox was prepared according to the literature [50,68], and IQ-1S was synthesized as described previously [53].

#### 3.1.1. General Procedure for Synthesis of Ketones **3a**–**3n**

A solution of corresponding isatin (1 mmol), corresponding isatoic anhydride (1 mmol), and triethylamine (5 mmol) was refluxed in ~10 mL of toluene for 1–4 h. Reaction progress was monitored with TLC using chloroform as the developing solvent. After a completion of the reaction, the mixture was cooled to the room temperature, and the precipitated product was filtered off and washed with methanol. The final products were purified by recrystallization from 1,4-dioxane.

*8-Nitroindolo[2,1-b]quinazolin-6,12-dione* (**3a**). Yield 38%, mp > 250 °C. ^1^H NMR (600 MHz, DMSO-*d*_6_) δ ppm: 7.80 (m, 1H), 8.01 (d, *J* = 3.6 Hz, 1H), 8.38 (d, *J* = 7.8 Hz, 1H), 8.57 (d, *J* = 2.4 Hz, 1H), 8.70 (d, *J* = 9.0 Hz, 1H), 8.74 (dd, *J*_1_ = 9.0 Hz, *J*_2_ = 2.4 Hz, 1H). ^13^C NMR (150 MHz, DMSO*-d*_6_) δ ppm: 118.15, 119.97, 123.30, 123.62, 127.71, 130.59, 130.83, 133.07, 136.27, 145.72, 146.11, 146.60, 149.64, 158.40, 181.32. C_15_H_7_N_3_O_4_. M.W. 293.23. Mass spectrum (EI) *m*/*z* (I, %): 293 (100), 265 (15), 219 (25).

*8-Bromoindolo[2,1-b]quinazolin-6,12-dione* (**3b**). Yield 42%, mp = 285 °C. ^1^H NMR (600 MHz, DMSO-*d*_6_) δ ppm: 7.76 (m, 1H), 7.97 (d, *J* = 3.6 Hz, 2H), 8.07 (m, 2H), 8.34 (d, *J* = 7.8 Hz, 1H), 8.43 (d, *J* = 8.4 Hz, 1H). ^13^C NMR (150 MHz, DMSO*-d*_6_) δ ppm: 119.41, 119.61, 123.59, 124.73, 127.47, 127.58, 130.46, 130.52, 135.82, 140.18, 145.27, 146.87, 158.11, 181.68. C_15_H_7_BrN_2_O_2_. M.W. 327.13. Mass spectrum (EI) *m*/*z* (I, %): 328 (800), 326 (100), 300 (30), 298 (30).

*8-Ethylindolo[2,1-b]quinazolin-6,12-dione* (**3c**). Yield 35%, mp = 221 °C. ^1^H NMR (600 MHz, DMSO-*d*_6_) δ ppm: 1.23 (t, *J* = 7.8 Hz, 3H), 2.72 (q, *J* = 7.8 Hz, 2H), 7.17 (m, 3H), 7.94 (m, 2H), 8.30 (d, *J* = 7.8 Hz, 1H), 8.36 (d, *J* = 8.4 Hz, 1H). ^13^C NMR (150 MHz, DMSO*-d*_6_) δ ppm: 15.92, 28.01, 117.36, 122.80, 123.79, 124.11, 127.33, 130.25, 130.34, 135.51, 137.63, 143.41, 144.59, 145.67, 146.92, 157.94, 182.94. C_17_H_12_N_2_O_2_. M.W. 276.29. Mass spectrum (EI) *m*/*z* (I, %): 276 (70), 261 (100), 233 (20).

*8-Methoxyindolo[2,1-b]quinazolin-6,12-dione* (**3d**). Yield 59%, mp = 265 °C. ^1^H NMR (600 MHz, DMSO*-d*_6_) δ ppm: 3.88 (s, 3H), 7.42 (m, 2H), 7.41 (m, 1H), 7.94 (d, *J* = 4.2 Hz, 2H), 8.31 (d, *J* = 7.8 Hz, 1H), 8.38 (d, *J* = 9.0 Hz, 1H). ^13^C NMR (150 MHz, DMSO*-d*_6_) δ ppm: 56.51, 108.95, 118.72, 123.86, 123.89, 124.22, 127.28, 130.30, 130.37, 135.44, 140.40, 145.85, 146.91, 157.77, 158.47, 182.83. C_16_H_10_N_2_O_3_. M.W. 278.26. Mass spectrum (EI) *m*/*z* (I, %): 278 (100), 263 (40), 235 (20).

*8-Phenylindolo[2,1-b]quinazolin-6,12-dione* (**3e**). Yield 65%, mp = 215–246 °C. ^1^H NMR (600 MHz, DMSO*-d*_6_) δ ppm: 7.43 (1H, dd, *J*_1_ = 7.3 Hz, *J*_2_ = 7.3 Hz), 7.51 (2H, dd, *J*_1_ = 8.1 Hz, *J*_2_ = 8.1 Hz), 7.74 (m, 1H), 7.79 (2H, d, *J* = 7.3 Hz), 7.95 (2H, m), 8.12 (1H, d, *J* = 2.1 Hz), 8.19 (1H, dd, *J*_1_ = 8.1 Hz, *J*_2_ = 2.1 Hz), 8.34 (1H, d, *J* = 7.7 Hz), 8.54 (1H, d, *J* = 8.3 Hz). ^13^C NMR (150 MHz, DMSO*-d*_6_) δ ppm: 117.5, 122.2, 122.3, 123.0, 123.3, 126.7, 126.9, 128.2, 129.1, 129.8, 135.2, 135.9, 138.1, 138.7, 145.1, 145.3, 146.4, 157.6, 182.4. C_21_H_12_N_2_O_2_. M.W. 324.33. Mass spectrum (EI) *m*/*z* (I, %): 324 (100), 296 (20).

*2-Bromo-8-nitroindolo[2,1-b]quinazolin-6,12-dione* (**3f**). Yield 29%, mp > 300 °C. ^1^H NMR (600 MHz, DMSO*-d*_6_) δ ppm: 7.95 (1H, d, *J* = 8.6 Hz), 8.17 (1H, dd, *J*_1_ = 8.6 Hz, *J*_2_ = 2.4 Hz), 8.44 (1H, d, *J* = 7.8 Hz), 8.56 (1H, d, *J* = 2.4 Hz), 8.66 (1H, d, *J* = 8.8 Hz), 8.73 (1H, dd, *J*_1_ = 8.8 Hz, *J*_2_ = 2.4 Hz). ^13^C NMR (150 MHz, DMSO*-d*_6_) δ ppm: 117.7, 119.5, 123.1, 123.3, 124.4, 129.4, 132.2, 132.6, 138.6, 145.2, 145.5, 145.8, 148.8, 156.7, 180.5. C_15_H_6_BrN_3_O_4_. M.W. 372.13. Mass spectrum (EI) *m*/*z* (I, %): 373 (100), 371 (100), 343 (10), 341 (10).

*7-Methylindolo[2,1-b]quinazolin-6,12-dione* (**3g**). Yield 61%, mp = 252–253 °C. ^1^H NMR (600 MHz, DMSO*-d*_6_) δ ppm: 7.52 (1H, dd, *J*_1_ = 7.5 Hz, *J*_2_ = 7.5 Hz), 7.91 (2H, m), 8.17 (1H, d, *J* = 8.8 Hz), 8.47 (1H, d, *J* = 8.1 Hz), 8.66 (1H, dd, *J*_1_ = 8.8 Hz, *J*_2_ = 2.6 Hz), 8.95 (1H, d, *J* = 2.6 Hz). ^13^C NMR (150 MHz, DMSO*-d*_6_) δ ppm: 122.4, 127.4, 127.6, 129.0, 130.3, 132.7, 134.4, 136.7, 143.2, 150.9, 152.3, 152.6, 155.9, 162.2, 187.2. C_16_H_10_N_2_O_2_. M.W. 262.26. Mass spectrum (EI) *m*/*z* (I, %): 262 (100), 234 (10).

*9-Methylindolo[2,1-b]quinazolin-6,12-dione* (**3h**). Yield 58%, mp = 255−256 °C. ^1^H NMR (600 MHz, DMSO*-d*_6_) δ ppm: 2.5 (3H, s), 7.26 (1H, d, *J* = 7.7 Hz), 7.71 (1H, m), 7.75 (1H, d, *J* = 7.7 Hz), 7.92 (2H, m), 8.28 (2H, m). ^13^C NMR (150 MHz, DMSO*-d*_6_) δ ppm: 22.4, 117.3, 117.4, 119.9, 123.2, 124.6, 126.9, 127.5, 129.7, 129.8, 135.1, 145.3, 146.3, 149.4, 157.6, 181.7. C_16_H_10_N_2_O_2_. M.W. 262.26. Mass spectrum (EI) *m*/*z* (I, %): 262 (100), 234 (30).

*2-Trifluoromethylindolo[2,1-b]quinazolin-6,12-dione* (**3i**). Yield 63%, mp = 357–358 °C. ^1^H NMR (600 MHz, DMSO*-d*_6_) δ ppm: 7.51 (1H, dd, *J*_1_ = 7.5 Hz, *J*_2_ = 7.5 Hz), 7.91 (2H, m), 8.16 (1H, d, *J* = 8.4 Hz), 8.27 (1H, dd, *J*_1_ = 2.0 Hz, *J*_2_ = 8.4 Hz), 8.47 (1H, d, *J* = 8.1 Hz), 8.54 (1H, d, *J* = 0.9 Hz). ^13^C NMR (150 MHz, DMSO*-d*_6_) δ ppm: 117.1, 122.1, 122.6, 123.7, 123.9, 124.4, 124.9, 127.2, 129.1, 131.2, 137.9, 145.7, 146.7, 149.1, 157.0, 182.1. ^19^F NMR (376 MHz, DMSO) δ ppm: −61.10 (s). C_16_H_7_F_3_N_2_O_2_. M.W. 316.23. Mass spectrum (EI) *m*/*z* (I, %): 316 (100), 288 (50), 266 (10).

*2-Bromoindolo[2,1-b]quinazolin-6,12-dione* (**3j**). Yield 46%, mp = 366–367 °C. ^1^H NMR (600 MHz, DMSO*-d*_6_) δ ppm: 7.49 (1H, dd, *J*_1_ = 7.6 Hz, *J*_2_ = 7.6 Hz), 7.88 (3H, m), 8.11 (1H, ddd, *J*_1_ = 8.6 Hz, *J*_2_ = 2.0 Hz, *J*_3_ = 2.0 Hz), 8.39 (1H, d, *J* = 2.0 Hz), 8.46 (1H, d, *J* = 8.6 Hz). ^13^C NMR (150 MHz, DMSO*-d*_6_) δ ppm: 117.6, 122.7, 123.3, 125.3, 125.4, 127.6, 129.6, 132.5, 138.3, 138.5, 145.9, 146.0, 146.2, 157.1, 182.7. C_15_H_7_BrN_2_O_2_. M.W. 327.13. Mass spectrum (EI) *m*/*z* (I, %): 328 (800), 326 (100), 300 (20), 298 (20).

*3-Bromoindolo[2,1-b]quinazolin-6,12-dione* (**3k**). Yield 59%, mp = 290–291 °C. ^1^H NMR (600 MHz, DMSO*-d*_6_) δ ppm: 7.49 (1H, dd, *J*_1_ = 4.5 Hz, *J*_2_ = 4.5 Hz), 7.88 (3H, m), 8.21 (2H, m), 8.45 (1H, d, *J* = 8.1 Hz), 7.48 (1H, dd, *J*_1_ = 7.5 Hz, *J*_2_ = 7.5 Hz). ^13^C NMR (150 MHz, DMSO*-d*_6_) δ ppm: 117.0, 122.1, 122.5, 124.9, 127.0, 128.6, 128.7, 132.0, 132.8, 137.9, 145.8, 146.1, 147.7, 157.3, 182.2. C_15_H_7_BrN_2_O_2_. M.W. 327.13. Mass spectrum (EI) *m*/*z* (I, %): 328 (100), 326 (95), 300 (30), 298 (30).

*3-Fluoroindolo[2,1-b]quinazolin-6,12-dione* (**3l**). Yield 53%, mp = 289 °C. ^1^H NMR (600 MHz, DMSO*-d*_6_) δ ppm: 7.48 (1H, dd, *J*_1_ = 7.5 Hz, *J*_2_ = 7.5 Hz), 7.6 (1H, ddd, *J*_1_ = 8.7 Hz, *J*_2_ = 8.7 Hz, *J*_3_ = 2.0 Hz), 7.81 (1H, dd, *J*_1_ = 9.7 Hz, *J*_2_ = 2.5 Hz), 7.88 (2H, m), 8.37 (1H, dd, *J*_1_ = 8.7 Hz, *J*_2_ = 6.1 Hz), 8.46 (1H, d, *J* = 7.9 Hz). ^13^C NMR (150 MHz, DMSO*-d*_6_) δ ppm: 115.3, 117.0, 118.0, 120.3, 122.1, 124.8, 127.0, 129.9, 137.8, 145.9, 148.7, 157.1, 165.0, 166.6, 182.2. ^19^F NMR (376 MHz, DMSO) δ ppm: −103.33 (s). C_15_H_7_FN_2_O_2_. M.W. 266.23. Mass spectrum (EI) *m*/*z* (I, %): 266 (100), 238 (70), 210 (40).

*3-Nitroindolo[2,1-b]quinazolin-6,12-dione* (**3m**). Yield 59%, mp > 300 °C. ^1^H NMR (600 MHz, DMSO*-d*_6_) δ ppm: 7.54 (1H, dd, *J*_1_ = 6.8 Hz, *J*_2_ = 6.8 Hz), 7.91 (2H, m), 8.45 (1H, dd, *J*_1_ = 8.6 Hz, *J*_2_ = 2.2 Hz), 8.49 (1H, d, *J* = 7.9 Hz), 8.56 (1H, d, *J* = 8.6 Hz), 8.67 (1H, d, *J* = 2.2 Hz). ^13^C NMR (150 MHz, DMSO*-d*_6_) δ ppm: 117.5, 119.5, 122.4, 127.4, 128.6, 129.7, 130.2, 132.9, 134.2, 137.4, 147.7, 150.9, 151.9, 152.3, 156.7. C_15_H_7_N_3_O_4_. M.W. 293.23. Mass spectrum (EI) *m*/*z* (I, %): 293 (100), 265 (20), 247 (10), 219 (10).

*2-Nitroindolo[2,1-b]quinazolin-6,12-dione* (**3n**). Yield 33%, mp > 300 °C. ^1^H NMR (600 MHz, DMSO*-d*_6_) δ ppm: 7.52 (1H, dd, *J*_1_ = 7.5 Hz, *J*_2_ = 7.5 Hz), 7.91 (2H, m), 8.17 (1H, d, *J* = 8.8 Hz), 8.47 (1H, d, *J* = 8.1 Hz), 8.66 (1H, dd, *J*_1_ = 8.8 Hz, *J*_2_ = 2.6 Hz), 8.95 (1H, d, *J* = 2.6 Hz). ^13^C NMR (150 MHz, DMSO*-d*_6_) δ ppm: 122.4, 127.4, 127.6, 129.0, 130.3, 132.7, 134.4, 136.7, 143.2, 150.9, 152.3, 152.6, 155.9, 162.2, 187.2. C_15_H_7_N_3_O_4_. M.W. 293.23. Mass spectrum (EI) *m*/*z* (I, %): 293 (100), 265 (20), 247 (10), 219 (25).

#### 3.1.2. General Procedure for Synthesis of Ketones **3o**–**3u**

Twenty mL of DMSO was added to the corresponding isatin (5 mmol), the mixture was heated to dissolve the isatin, and potassium phosphate (5 mmol) was added. After cooling, tert-butyl hydroperoxide (5 mmol, 550 μL of 70% aqueous solution) was added, and the mixture was left at room temperature for at least 20 h. The progress of the reaction was monitored with TLC, using chloroform as the developing solvent. The reaction mixture was poured into 100 mL of water and left to precipitate overnight. The precipitate was filtered off, washed with water (2 × 5 mL), and dried at 105 °C. The final products were purified by recrystallization from 1,4-dioxane.

*2,8-Dimethylindolo[2,1-b]quinazolin-6,12-dione* (**3o**). Yield 20%, mp = 236–237 °C. ^1^H NMR (600 MHz, DMSO*-d*_6_) δ ppm: 2.39 (3H, s), 2.51 (3H, s), 7.65 (2H, m), 7.74 (1H, dd, *J*_1_ = 8.2 Hz, *J*_2_ = 1.8 Hz), 7.82 (1H, d, *J* = 8.3 Hz), 8.09 (1H, d, *J* = 1.8 Hz), 8.33 (1H, d, *J* = 8.1 Hz). ^13^C NMR (150 MHz, DMSO*-d*_6_) δ ppm: 20.4, 21.0, 116.8, 122.3, 123.1, 124.7, 126.4, 129.7, 136.1, 136.6, 138.0, 140.2, 143.9, 144.4, 144.5, 157.4, 182.4. C_17_H_12_N_2_O_2_. M.W. 276.30. Mass spectrum (EI) *m*/*z* (I, %): 276 (100), 248 (30)

*1,7-Dimethylindolo[2,1-b]quinazolin-6,12-dione* (**3p**). Yield 8%, mp = 290–291 °C. ^1^H NMR (600 MHz, DMSO*-d*_6_) δ ppm: 2.62 (s, 3H), 2.87 (3H, s), 7.24 (1H, d, *J* = 7.7 Hz), 7.47 (1H, m), 7.68 (1H, dd, *J*_1_ = 7.8 Hz, *J*_2_ = 7.8 Hz), 7.74 (2H, m), 8.32 (1H, d, *J* = 8.1 Hz). ^13^C NMR (150 MHz, DMSO*-d*_6_) δ ppm: 17.7, 22.6, 114.5, 119.9, 121.4, 128.2, 128.6, 132.4, 134.0, 136.9, 139.7, 141.4, 144.5, 146.3, 148.0, 158.5, 183.1. C_17_H_12_N_2_O_2_. M.W. 276.30. Mass spectrum (EI) *m*/*z* (I, %): 276 (100), 248 (20).

*2,8-Dimethoxylindolo[2,1-b]quinazolin-6,12-dione* (**3q**). Yield 29%, mp = 260–261 °C. ^1^H NMR (600 MHz, DMSO*-d*_6_) δ ppm: 3.86 (3H, s), 3.95 (3H, s), 7.38 (1H, d, *J* = 2.7 Hz), 7.42 (1H, dd, *J*_1_ = 8.6 Hz, *J*_2_ = 2.7 Hz), 7.51 (1H, dd, *J*_1_ = 8.8 Hz, *J*_2_ = 2.9 Hz), 7.7 (1H, d, *J* = 3.0 Hz), 7.87 (1H, d, *J* = 9.0 Hz), 8.37 (1H, d, *J* = 8.7 Hz). ^13^C NMR (150 MHz, DMSO*-d*_6_) δ ppm: 61.2, 113.4, 113.6, 123.5, 128.6, 128.9, 130.1, 136.9, 137.0, 145.0, 145.7, 148.8, 162.1, 163.2, 165.6, 187.4. C_17_H_12_N_2_O_4_. M.W. 308.30. Mass spectrum (EI) *m*/*z* (I, %): 308 (100), 293 (40).

*3,9-Dimethoxylindolo[2,1-b]quinazolin-6,12-dione* (**3r**). Yield 62%, mp > 300 °C. ^1^H NMR (600 MHz, DMSO*-d*_6_) δ ppm: 3.94 (3H, s), 3.97 (3H, s), 6.97 (1H, dd, *J*_1_ = 8.4 Hz, *J*_2_ = 2.2 Hz), 7.29 (1H, dd, *J*_1_ = 8.8 Hz, *J*_2_ = 2.5 Hz), 7.42 (1H, d, *J* = 2.5 Hz), 7.82 (1H, d, *J* = 8.5 Hz), 7.99 (1H, d, *J* = 2.2 Hz), 8.2 (1H, d, *J* = 8.8 Hz). ^13^C NMR (150 MHz, DMSO*-d*_6_) δ ppm: 55.9, 56.4, 103.0, 111.9, 113.6, 115.1, 116.6, 119.0, 127.3, 128.8, 146.0, 148.9, 149.1, 157.9, 165.0, 168.0, 180.3. C_17_H_12_N_2_O_4_. M.W. 308.30. Mass spectrum (EI) *m*/*z* (I, %): 308 (100).

*3,9-Dibromoindolo[2,1-b]quinazolin-6,12-dione* (**3s**). Yield 38%, mp = 350–351 °C. ^1^H NMR (600 MHz, DMSO*-d*_6_) δ ppm: 7.72 (1H, dd, *J*_1_ = 8.1 Hz, *J*_2_ = 1.6 Hz), 7.84 (1H, d, *J* = 8.1 Hz), 7.93 (1H, dd, *J*_1_ = 8.4 Hz, *J*_2_ = 1.8 Hz), 8.22 (2H, m), 8.6 (1H, d, *J* = 1.7 Hz). ^13^C NMR (150 MHz, DMSO*-d*_6_) δ ppm: 120.2, 121.9, 122.6, 126.8, 129.3, 129.4, 130.5, 131.4, 132.6, 133.4, 146.4, 146.9, 148.1, 157.9, 181.7. C_15_H_6_Br_2_N_2_O_2_. M.W. 406.04. Mass spectrum (EI) *m*/*z* (I, %): 404/406/408 (95/100/95), 376/378/380 (20/30/20).

*2,8-Dibromoindolo[2,1-b]quinazolin-6,12-dione* (**3t**). Yield 25%, mp = 310–311 °C. ^1^H NMR (600 MHz, DMSO*-d*_6_) δ ppm: 7.91 (1H, d, *J* = 8.4 Hz), 8.05 (1H, dd, *J*_1_ = 8.4 Hz, *J*_2_ = 2.0 Hz), 8.08 (1H, d, *J* = 2.0 Hz), 8.13 (1H, dd, *J*_1_ = 8.5 Hz, *J*_2_ = 2.3 Hz), 8.38 (1H, d, *J* = 8.7 Hz), 8.41 (1H, d, *J* = 2.4 Hz). ^13^C NMR (150 MHz, DMSO*-d*_6_) δ ppm: 118.9, 119.4, 123.0, 124.2, 124.7, 127.1, 129.1, 132.0, 138.2, 139.7, 144.5, 145.1, 145.5, 156.5, 180.9. C_15_H_6_Br_2_N_2_O_2_. M.W. 406.04. Mass spectrum (EI) *m*/*z* (I, %): 404/406/408 (95/100/95), 376/378/380 (15/20/15).

*1,7-Dibromoindolo[2,1-b]quinazolin-6,12-dione* (**3u**). Yield 21%, mp > 300 °C. ^1^H NMR (600 MHz, DMSO*-d*_6_) δ ppm: 7.67 (1H, d, *J* = 8.2 Hz), 7.73 (1H, dd, *J*_1_ = 7.9 Hz, *J*_2_ = 7.9 Hz), 7.78 (1H, dd, *J*_1_ = 7.9 Hz, *J*_2_ = 7.9 Hz), 7.96 (2H, m), 8.52 (1H, d, *J* = 7.9 Hz). ^13^C NMR (150 MHz, DMSO*-d*_6_) δ ppm: 116.2, 119.6, 120.5, 121.0, 121.5, 130.2, 131.2, 135.4, 136.2, 138.3, 144.7, 147.6, 148.9, 155.9, 179.9. C_15_H_6_Br_2_N_2_O_2_. M.W. 406.04. Mass spectrum (EI) *m*/*z* (I, %): 404/406/408 (95/100/95), 376/378/380 (15/20/15).

#### 3.1.3. General Procedure for Synthesis of Compounds **4a**–**u**

Hydroxylamine hydrochloride (3 mmol) was added to a solution of 1 mmol of the substituted ketones (**3a**–**u**) in 6 mL of pyridine, and the mixture was refluxed for 4 h. The progress of the reaction was monitored with TLC, using chloroform–methanol mixture (10/1) as the developing solvent. The excess pyridine was removed under reduced pressure, and the residue was poured into a 0.1 M HCl solution. The precipitate was filtered off, washed with water (2 × 5 mL), and recrystallized from 1,4-dioxane.

*8-Nitroindolo[2,1-b]quinazolin-6,12-dione Oxime* (**4a**). Yield 95%, mp = 227–228 °C. ^1^H NMR (600 MHz, DMSO*-d*_6_) δ ppm: 7.67 (ddd, *J*_1_ = 7.2 Hz, *J*_2_ = 7.2 Hz, *J*_3_ = 0.6 Hz, 1H), 7.86 (d, *J* = 8.4 Hz, 1H), 7.94 (ddd, *J*_1_ = 7.2 Hz, *J*_2 =_ 7.2, J_3_ = 1.8 Hz, 1H), 8.33 (d, *J* = 7.2 Hz, 1H), 8.59 (dd, *J*_1_ = 9.0 Hz, *J*_2_ = 2.2Hz, 1H), 8.73 (d, *J* = 8.4Hz, 1H), 9.05 (d, *J* = 2.4Hz, 1H), 14.18 (s, 1H). ^13^C NMR (150 MHz, DMSO*-d*_6_) δ ppm: 117.19, 119.86, 121.81, 122.33, 127.28, 128.47, 128.76, 135.76, 143.68, 143.98, 145.57, 147.25, 148.74, 150.09, 159.18. C_15_H_8_N_4_O_4_. M.W. 308.25. Mass spectrum (EI) *m*/*z* (I, %): 308 (100), 232 (20).

*8-Bromoindolo[2,1-b]quinazolin-6,12-dione Oxime* (**4b**). Yield 90%, mp > 225 °C (dec). ^1^H NMR (600 MHz, DMSO*-d*_6_) δ ppm: 7.64 (dd, *J*_1_ = 7.8 Hz, *J*_2_ = 7.8 Hz, 1H), 7.83 (d, *J* = 7.8 Hz, 1H), 7.88 (dd, *J*_1_ = 8.4 Hz, *J*_2_ = 2.4 Hz, 1H), 7.91 (ddd, *J*_1_ = 7.8 Hz, *J*_2_ = 7.5 Hz, *J*_3_ = 1.8 Hz, 1H), 8.30 (dd, *J*_1_ = 7.8 Hz, *J*_2_ = 1.8 Hz, 1H), 8.47 (m, 2H), 13.93 (s, 1H). ^13^C NMR (150 MHz, DMSO*-d*_6_) δ ppm: 118.60, 118.99, 121.14, 121.96, 127.07, 128.20, 128.67, 129.90, 135.03, 135.35, 138.91, 143.87, 147.45, 148.42, 158.91. C_15_H_8_BrN_3_O_2_. M.W. 342.15. Mass spectrum (EI) *m*/*z* (I, %): 343 (100), 341 (100), 313 (50), 311 (50).

*8-Ethylindolo[2,1-b]quinazolin-6,12-dione Oxime* (**4c**). Yield 95%, mp > 225 °C (dec). ^1^H NMR (600 MHz, DMSO*-d*_6_) δ ppm: 1.23 (s, CH_3_, *J* = 7.8 Hz, 3H), 2.72 (q, CH_2_, *J* = 7.8 Hz, 2H), 7.50 (d, *J* = 7.8 Hz, 1H), 7.62 (dd, *J*_1_ = 7.8 Hz, *J*_2_ = 7.8 Hz, 1H), 7.80 (d, *J* = 8.4 Hz, 1H), 7.83 (dd, *J*_1_ = 7.2 Hz, *J*_2_ = 7.2 Hz, 1H), 8.22 (s, 1H), 8.29 (d, *J* = 7.8 Hz, 1H), 8.43 (d, *J* = 8.4 Hz, 1H), 13.62 (s, 1H). ^13^C NMR (150 MHz, DMSO*-d*_6_) δ ppm: 16.24, 28.48, 116.59, 119.50, 122.06, 126.98, 127.09, 127.95, 128.58, 131.88, 135.08, 137.86, 142.96, 144.75, 147.48, 148.98, 158.76. C_17_H_13_N_3_O_2_. M.W. 291.30. ESI-MS: 268 (40%), 266 (100%), 238 (30%). ESI-MS: [M + H]^+^ Calcd for C_17_H_13_N_3_O_2_ 292.1086; Found 292.1074.

*8-Methoxyindolo[2,1-b]quinazolin-6,12-dione Oxime* (**4d**). Yield 85%, mp > 225 °C (dec). ^1^H NMR (600 MHz, DMSO*-d*_6_) δ ppm: 3.84 (s, CH_3_, 3H), 7.21 (dd, *J*_1_ = 8.4 Hz, *J*_2_ = 2.4 Hz, 1H), 7.60 (ddd, *J*_1_ = 7.8 Hz, *J*_2_ = 7.8 Hz, *J*_3_ = 1.2 Hz, 1H), 7.80 (d, *J* = 7.8 Hz, 1H), 7.87 (m, 2H), 8.27 (dd, *J*_1_ = 7.8 Hz, *J*_2_ = 1.8 Hz, 1H), 8.42 (d, *J* = 9.0 Hz, 1H), 13.70 (s, 1H). ^13^C NMR (150 MHz, DMSO*-d*_6_) δ ppm: 56.17, 113.01, 117.67 (2C), 120.34, 122.06, 126.88, 127.93, 128.57, 133.44, 134.96, 144.65, 147.41, 148.92, 158.06, 158.51. ESI-MS: [M + H]^+^ Calcd for C_16_H_11_N_3_O_3_ 294.0878; Found 294.0872.

*8-Phenylindolo[2,1-b]quinazolin-6,12-dione Oxime* (**4e**). Yield 67%, mp > 225 °C (dec). ^1^H NMR (600 MHz, DMSO*-d*_6_) δ ppm: 7.43 (dd, *J*_1_ = 7.4 Hz, *J*_2_ = 7.4 Hz, 1H), 7.52 (dd, *J*_1_ = 7.5 Hz, *J*_2_ = 7.5 Hz, 2H), 7.63 (ddd, *J*_1_ = 7.5 Hz, *J*_2_ = 7.5 Hz, *J*_3_ = 1.0 Hz, 1H), 7.70 (d, *J* = 7.2 Hz, 2H), 7.83 (d, *J* = 7.7 Hz, 1H), 7.90 (ddd, *J*_1_ = 7.6 Hz, *J*_2_ = 7.6 Hz, *J*_3_ = 1.5 Hz, 1H), 7.95 (dd, *J*_1_ = 8.4 Hz, *J*_2_ = 1.9 Hz, 1H), 8.30 (dd, 1H, *J*_1_ = 7.8 Hz, *J*_2_ = 1.3 Hz), 8.59 (m, 2H), 13.72 (s, 1H). ^13^C NMR (150 MHz, DMSO*-d*_6_) δ ppm: 117.14, 120.09, 122.03, 125.75, 127.04, 127.23 (2C), 128.06, 128.33, 128.63, 129.62 (2C), 131.01, 135.21, 139.05, 139.15, 139.70, 144.65, 147.46, 148.93, 158.90. ESI-MS: [M + H]^+^ Calcd for C_21_H_13_N_3_O_2_ 340.1086; Found 340.1090.

*2-Bromo-8-nitroindolo[2,1-b]quinazolin-6,12-dione Oxime* (**4f**). Yield 36%, mp > 225 °C (dec). ^1^H NMR (600 MHz, DMSO*-d*_6_) δ ppm: 7.80 (d, *J* = 8.7 Hz, 1H), 8.09 (dd, *J*_1_ = 8.6 Hz, *J*_2 =_ 2.4 Hz, 1H), 8.39 (d, *J* = 2.2 Hz, 1H), 8.59 (dd, *J*_1_ = 8.9 Hz, *J*_2_ = 2.5 Hz, 1H), 8.70 (d, *J* = 8.8 Hz, 1H), 9.04 (d, *J* = 2.4 Hz, 1H), 14.28 (s, 1H). ^13^C NMR (150 MHz, DMSO*-d*_6_) δ ppm: 117.29, 119.83, 120.98, 122.30, 123.50, 128.52, 128.68, 129.30, 130.97, 138.49, 143.66, 145.75, 146.32, 149.24, 158.07. ESI-MS: [M + H]^+^ Calcd for C_15_H_7_BrN_4_O_4_ 386.9728; Found 386.9726.

*7-Methylindolo[2,1-b]quinazolin-6,12-dione Oxime* (**4g**). Yield 61%, mp > 225 °C (dec). ^1^H NMR (600 MHz, DMSO*-d*_6_) δ ppm: 3.32 (s, 3H), 7.25 (d, *J*_1_ = 7.5 Hz, 1H), 7.47 (dd, *J*_1_ = *J*_2_ = 7.8 Hz, 1H), 7.68 (ddd, *J*_1_ = *J*_2_ = 5.8 Hz, *J*_3_ = 1.5 Hz, 1H), 7.90 (m, 2H), 8.32 (dd, *J*_1_ = 7.8 Hz, *J*_2_ = 0.9 Hz, 1H), 8.39 (d, *J* = 8.0 Hz, 1H), 13.71 (s,1H). ^13^C NMR (150 MHz, DMSO*-d*_6_) δ ppm: 20.86, 114.37, 120.77, 122.18, 126.97, 128.85, 128.93, 128.99, 130.66, 135.05, 135.15, 139.48, 143.50, 144.87, 147.36, 158.66. ESI-MS: [M + H]^+^ Calcd for C_16_H_11_N_3_O_2_ 278.0929; Found 278.0929.

*9-Methylindolo[2,1-b]quinazolin-6,12-dione Oxime* (**4h**). Yield 66%, mp > 225 °C (dec). ^1^H NMR (600 MHz, DMSO*-d*_6_) δ ppm: 2.47 (s, 3H), 7.26 (dd, *J*_1_ = 7.7 Hz, *J*_2_ = 1.1 Hz, 1H), 7.62 (ddd, *J*_1_ = 7.6 Hz, *J*_2_ = 7.6 Hz, *J*_3_ = 0.6 Hz, 1H), 7.81 (d, *J* = 7.5 Hz, 1H), 7.89 (ddd, *J*_1_ = 7.6 Hz, *J*_2_ = 7.6 Hz, *J*_3_ = 1.7 Hz, 1H), 8.24 (d, *J* = 7.7 Hz, 1H), 8.29 (dd, *J*_1_ = 7.8 Hz, *J*_2_ = 1.3 Hz, 1H), 8.38 (s, 1H), 13.48 (s, 1H). ^13^C NMR (150 MHz, DMSO*-d*_6_) δ ppm: 22.41, 117.02, 117.24, 122.04, 127.02, 127.66 (2C), 128.00, 128.58, 135.17, 140.06, 143.03, 144.62, 147.42, 149.12, 158.95. ESI-MS: [M + H]^+^ Calcd for C_16_H_11_N_3_O_2_ 278.0929; Found 278.0933.

*2-Trifluoromethylindolo[2,1-b]quinazolin-6,12-dione Oxime* (**4i**). Yield 64%, mp > 225 °C (dec). ^1^H NMR (600 MHz, DMSO*-d*_6_) δ ppm: 7.48 (m, 1H), 7.67 (m, 1H), 7.99 (m, 1H), 8.18 (m, 1H), 8.36 (m, 1H), 8.49 (m, 2H), 13.86 (s, 1H). ^13^C NMR (150 MHz, DMSO*-d*_6_) δ ppm: 116.82, 119.20, 122.31, 123.35, 124.21, 125.15, 127.51, 127.81, 130.00, 131.10, 132.65, 139.46, 144.67, 150.08, 150.98, 158.33. ^19^F NMR (376 MHz, DMSO) δ ppm: −60.88 (s). ^19^F NMR (^1^H-decoupled) (376 MHz, DMSO) δ ppm: −60.88 (s). ESI-MS: [M + H]^+^ Calcd for C_16_H_8_F_3_N_3_O_2_ 332.0646; Found 332.0648.

*2-Bromoindolo[2,1-b]quinazolin-6,12-dione Oxime* (**4j**). Yield 43%, mp > 225 °C (dec). ^1^H NMR (600 MHz, DMSO*-d*_6_) δ ppm: 7.46 (dd, *J*_1_ = 7.5 Hz, *J*_2_ = 7.5 Hz, 1H), 7.66 (dd, *J*_1_ = 7.5 Hz, *J*_2_ = 7.5 Hz, 1H), 7.75 (d, *J* = 8.7 Hz, 1H), 8.02 (dd, *J*_1_ = 8.6 Hz, *J*_2_ = 2.3 Hz, 1H), 8.34 (d, *J* = 2.4 Hz, 1H), 8.37 (d, *J* = 7.6 Hz, 1H), 8.50 (d, *J* = 8.0 Hz, 1H), 13.72 (s, 1H). ^13^C NMR (150 MHz, DMSO*-d*_6_) δ ppm: 116.78, 119.31, 120.49, 123.72, 127.38, 127.85, 129.08, 130.82, 132.60, 137.90, 139.53, 144.62, 146.52, 149.31, 157.83. ESI-MS: [M + H]^+^ Calcd for C_15_H_8_BrN_3_O_2_ 341.9878; Found 341.9877.

*3-Bromoindolo[2,1-b]quinazolin-6,12-dione Oxime* (**4k**). Yield 60%, mp > 225 °C (dec). ^1^H NMR (600 MHz, DMSO*-d*_6_) δ ppm: 7.47 (dd, *J*_1_ = 7.6 Hz, *J*_2_ = 7.6 Hz, 1H), 7.66 (dd, *J*_1_ = 7.9 Hz, *J*_2_ = 7.9 Hz, 1H), 7.78 (dd, *J*_1_ = 8.4 Hz, *J*_2_ = 1.7 Hz, 1H), 8.02 (s, 1H), 8.19 (d, *J* = 8.5 Hz, 1H), 8.37 (d, *J* = 7.5 Hz, 1H), 8.52 (d, *J* = 8.1 Hz, 1H), 13.75 (s, 1H). ^13^C NMR (150 MHz, DMSO*-d*_6_) δ ppm: 116.76, 119.25, 121.27, 127.33, 127.83, 128.63, 128.96, 130.74, 130.88, 132.63, 139.64, 144.65, 148.69, 150.12, 158.57. ESI-MS: [M + H]^+^ Calcd for C_15_H_8_BrN_3_O_2_ 341.9878; Found 341.9940.

*3-Fluoroindolo[2,1-b]quinazolin-6,12-dione Oxime* (**4l**). Yield 76%, mp > 225 °C (dec). ^1^H NMR (600 MHz, DMSO*-d*_6_) δ ppm: 7.46 (m, 2H), 7.60 (dd, *J*_1_ = 9.8 Hz, *J*_2_ = 2.1 Hz, 1H), 7.65 (dd, *J*_1_ = 7.8 Hz, *J*_2_ = 7.8 Hz, 1H), 8.32 (dd, *J*_1_ = 8.7 Hz, *J*_2_ = 7.5 Hz, 1H), 8.35 (d, *J* = 7.7 Hz, 1H), 8.49 (d, *J* = 8.1 Hz, 1H), 13.72 (s, 1H). ^13^C NMR (150 MHz, DMSO*-d*_6_) δ ppm: 113.87, 116.37, 116.72, 119.17, 127.23, 127.81, 129.95, 132.57, 139.64, 144.64, 149.67, 150.16, 158.30, 165.37, 167.04. ^19^F NMR (376 MHz, DMSO) δ ppm: −104.09 (m). ^19^F NMR (^1^H-decoupled) (376 MHz, DMSO) δ ppm: −104.10 (s). ESI-MS: [M + H]^+^ Calcd for C_15_H_8_FN_3_O_2_ 282.0678; Found 282.0681.

*3-Nitroindolo[2,1-b]quinazolin-6,12-dione Oxime* (**4m**). Yield 70%, mp > 225 °C (dec). ^1^H NMR (600 MHz, DMSO*-d*_6_) δ ppm: 7.50 (dd, *J*_1_ = 7.3 Hz, *J*_2_ = 7.3 Hz, 1H), 7.70 (ddd, *J*_1_ = 8.0 Hz, *J*_2_ = 8.0 Hz, *J*_3_ = 1.2 Hz, 1H), 8.33 (dd, *J*_1_ = 8.8 Hz, *J*_2_ = 2.2 Hz, 1H), 8.39 (d, *J* = 7.7 Hz, 1H), 8.52 (m, 3H), 13.89 (s, 1H). ^13^C NMR (150 MHz, DMSO*-d*_6_) δ ppm: 116.91, 119.28, 121.42, 123.38, 126.53, 127.68, 127.82, 129.15, 132.71, 139.45, 144.54, 147.99, 150.89, 151.65, 158.05. ESI-MS: [M + H]^+^ Calcd for C_15_H_8_N_4_O_4_ 309.0623; Found 309.0625.

*2-Nitroindolo[2,1-b]quinazolin-6,12-dione Oxime* (**4n**): Yield 55%, mp > 225 °C (dec). ^1^H NMR (600 MHz, DMSO*-d*_6_) δ ppm: 7.47 (ddd, *J*_1_ = 9.0 Hz, *J*_2_ = 8.0 Hz, *J*_3_ = 0.9 Hz, 1H), 7.66 (ddd, *J*_1_ = 9.0 Hz, *J*_2_ = 8.0 Hz, *J*_3_ = 1.3 Hz, 1H), 7.96 (d, *J* = 9.0 Hz, 1H), 8.34 (d, *J* = 7.2 Hz, 1H), 8.47 (d, *J* = 7.8 Hz, 1H), 8.57 (dd, *J*_1_ = 7.2 Hz, *J*_2_ = 2.4 Hz, 1H), 8.89 (d, *J* = 6.0 Hz 1H), 13.97 (s, 1H). ^13^C NMR (150 MHz, DMSO*-d*_6_) δ ppm: 116.83, 119.09, 122.27, 122.75, 127.67, 127.74, 128.94, 130.19, 132.66, 139.21, 144.62, 145.73, 151.68, 151.80, 158.09. ESI-MS: [M + H]^+^ Calcd for C_15_H_8_N_4_O_4_ 309.0623; Found 309.0622.

*2,8-Dimethylindolo[2,1-b]quinazolin-6,12-dione Oxime* (**4o**). Yield 58%, mp > 300 °C (dec). ^1^H NMR (600 MHz, DMSO*-d*_6_) δ ppm: 2.60 (s, 3H); 2.90 (s, 3H); 7.25 (d, *J* = 7.7 Hz, 1H); 7.43 (d, *J* = 7.7 Hz, 1H); 7.46 (dd, *J*_1_ = *J*_2_ = 7.9 Hz, 1H); 7.695 (d, *J* = 7.9 Hz, 1H); 7.74 (dd, *J*_1_ = *J*_2_ = 7.7 Hz, 1H); 8.40 (d, *J* = 8.0 Hz, 1H); 13.67 (s, 1H). ^13^C NMR (600 MHz, DMSO*-d*_6_) δ ppm: 66.8, 114.5, 120.5, 120.7, 127.3, 128.8, 130.7, 131.5, 134.2, 135.0, 139.7, 141.4, 143.4, 144.9, 148.9, 159.5. ESI-MS: [M + H]^+^ Calcd for C_17_H_13_N_3_O_2_ 292.1086; Found 292.1086.

*1,7-Dimethylindolo[2,1-b]quinazolin-6,12-dione Oxime* (**4p**). Yield 59%, mp = 190–191 °C. ^1^H NMR (600 MHz, DMSO*-d*_6_) δ ppm: 2.60 (s, 3H); 2.90 (s, 3H); 7.25 (d, *J* = 7.7 Hz, 1H); 7.43 (d, *J* = 7.7 Hz, 1H); 7.46 (dd, *J*_1_ = *J*_2_ = 7.9 Hz, 1H); 7.695 (d, *J* = 7.9 Hz, 1H); 7.74 (dd, *J*_1_ = *J*_2 =_ 7.7 Hz, 1H); 8.40 (d, *J* = 8.0 Hz, 1H);13.67 (s, 1H). ^13^C NMR (600 MHz, DMSO*-d*_6_) δ ppm: 66.8, 114.5, 120.5, 120.7, 127.3, 128.8, 130.7, 131.5, 134.2, 135.0, 139.7, 141.4, 143.4, 144.9, 148.9, 159.5. ESI-MS: [M + H]^+^ Calcd for C_17_H_13_N_3_O_2_ 292.1086; Found 292.1088.

*2,8-Dimethoxylindolo[2,1-b]quinazolin-6,12-dione Oxime* (**4q**). Yield 85%, mp = 262–263 °C. ^1^H NMR (600 MHz, DMSO*-d*_6_) δ ppm: 3.844 (s, 1H); 3.9 (s, 1H); 7.22 (dd, *J*_1_ = 2.7 Hz, *J*_2_ = 8.8 Hz, 1H); 7.465 (dd, *J*_1_ = 2.9 Hz, *J*_2_ = 8.8 Hz, 1H); 7.650 (d, *J* = 2.9 Hz, 1H); 7.743 (d, *J* = 8.8 Hz, 1H); 7.890 (d, *J* = 2.7 Hz, 1H); 8.430 (d, *J* = 8.8 Hz, 1H); 13.6 (s, 1H). ^13^C NMR (DMSO*-d*_6_) δ ppm: 56.2, 107.5, 113.1, 117.7, 120.5, 123.0, 124.0, 130.3, 133.4, 141.7, 144.5, 146.8, 158.1, 158.2, 159.0. ESI-MS: [M + H]^+^ Calcd for C_17_H_13_N_3_O_4_ 324.0984; Found 324.0922.

*3,9-Dimethoxylindolo[2,1-b]quinazolin-6,12-dione Oxime* (**4r**). Yield 30%, mp = 292–293 °C. ^1^H NMR (600 MHz, DMSO*-d*_6_) δ ppm: 3.90 (s, 3H); 3.935 (s, 3H); 6.98 (dd, *J*_1_ = 2.4 Hz, *J*_2_ = 8.8 Hz, 1H); 7.190 (dd, *J*_1_ = 2.5 Hz, *J*_2_ = 8.7 Hz, 1H); 7.268 (d, *J* = 2.5 Hz, 1H); 8.093 (d, *J* = 2.4 Hz, 1H); 8.165 (d, *J* = 8.8 Hz, 1H); 8.257 (d, *J* = 8.7 Hz, 1H); 13.287 (s, 1H). ^13^C NMR (600 MHz, DMSO*-d*_6_) δ ppm: 56.3, 56.4, 103.0, 110.2, 112.0, 112.5, 115.2, 117.2, 128.6, 129.2, 141.6, 144.2, 149.7, 150.0, 158.7, 162.4, 164.8. ESI-MS: [M + H]^+^ Calcd for C_17_H_13_N_3_O_4_ 324.0984; Found 324.0983.

*3,9-Dibromoindolo[2,1-b]quinazolin-6,12-dione Oxime* (**4s**). Yield 40%, mp > 300 °C (dec). ^1^H NMR (600 MHz, DMSO*-d*_6_) δ ppm: 7.68 (dd, *J*_1_ = 2.0 Hz, *J*_2_ = 8.4 Hz, 1H); 7.79 (dd, *J*_1_ = 2.0 Hz, *J*_2_ = 8.4 Hz, 1H); 8.034 (d, *J* = 1.8 Hz, 1H); 8.185 (d, *J* = 8.4 Hz, 1H); 8.27 (d, *J* = 8.4 Hz, 1H); 8.645 (d, *J* = 1.8 Hz, 1H); 13.91 (s, 1H). ^13^C NMR (600 MHz, DMSO*-d*_6_) δ ppm: 118.4, 119.4, 121.0, 125.1, 128.7, 129.0, 129.2, 129.4, 130.1, 130.8, 131.1, 140.5, 144.1, 148.6, 158.6. ESI-MS: [M − H]^−^ Calcd for C_15_H_7_Br_2_N_3_O_2_ 417.8826; Found 417.8880.

*2,8-Dibromoindolo[2,1-b]quinazolin-6,12-dione Oxime* (**4t**). Yield 56%, mp = 250–251 °C. ^1^H NMR (600 MHz, DMSO*-d*_6_) δ ppm: 7.773 (d, *J* = 8.6 Hz, 1H); 7.883 (dd, *J*_1_ = 2.1 Hz, *J*_2_ = 8.6 Hz, 1H); 8.055 (dd, *J*_1_ = 2.4 Hz, *J*_2_ = 8.7 Hz, 1H); 8.36 (d, *J* = 2.4 Hz, 1H); 8.45 (d, *J* = 8.7 Hz, 1H); 8.475 (d, *J* = 2.1 Hz, 1H); 14.022 (s, 1H). ^13^C NMR (DMSO*-d*_6_) δ ppm: 118.6, 119.3, 120.7, 121.1, 123.6, 129.1, 129.8, 130.1, 135.1, 138.1, 138.6, 143.8, 146.5, 148.9, 157.8. ESI-MS: [M − H]^−^ Calcd for C_15_H_7_Br_2_N_3_O_2_ 417.8826; Found 417.8833.

*1,7-Dibromoindolo[2,1-b]quinazolin-6,12-dione Oxime* (**4u**). Yield 21%, *mp* > 300 °C (dec). ^1^H NMR (600 MHz, DMSO*-d*_6_) δ ppm: 7.495 (dd, *J*_1_ = *J*_2_ = 8.0 Hz, 1H); 7.665 (dd, *J*_1_ = 0.7 Hz, *J*_2_ = 8.0 Hz, 1H); 7.735 (dd, *J*_1_ = *J*_2_ = 8.0 Hz, 1H); 7.860 (dd, *J*_1_ = 1.1 Hz; *J*_2_ = 8.0 Hz, 1H); 7.895 (dd, *J*_1_ = 1.1 Hz; *J*_2_ = 8.0 Hz, 1H); 8.575 (d, *J* = 0.7 Hz, 1H); 14.03 (s, 1H). ^13^C NMR (600 MHz, DMSO*-d*_6_) δ ppm: 115.5, 116.1, 119.9, 121.4, 121.8, 129.4, 131.6, 132.0, 135.2, 135.3, 141.0, 142.7, 143.1, 150.0, 157.0. ESI-MS: [M − H]^−^ Calcd for C_15_H_7_Br_2_N_3_O_2_ 417.8826; Found 417.8957.

#### 3.1.4. General Procedure for Synthesis of Compounds **5a**,**b**

A solution of monochloroacetic acid ethyl ester for **5a** (1.5 mmol in 5 mL of DMSO) or bromomalonic acid diethyl ester for **5b** (1.5 mmol in 5 mL of DMSO) was added to a suspension of TRYP-Ox (1.0 mmol) and KOH (2.0 mmol) in 5 mL of DMSO. The mixtures were stirred for 1 h at room temperature and added to 150 mL of water. The precipitates were filtered off and recrystallized from EtOH to obtain **5a** (69% yield) and **5b** (33% yield), respectively.

*Ethyl 2-(((12-Oxoindolo[2,1-b]quinazolin-6(12H)-ylidene)amino)oxy)acetate* (**5a**). Yield 69%, colorless crystals, mp = 218 °C. ^1^H NMR (400 MHz, CDCl_3_), δ, ppm: 1.34 (t, 3H, *J* = 6 Hz, CH_3_), 4.30 (q, 2H, *J* = 6 Hz, CH_2_CH_3_), 5.16 (s, 2H, =N-O-CH_2_), 7.40 (td, 1H, *J*_1_ = 1 Hz, *J*_2_ = 7.7 Hz), 7.56–7.65 (m, 2H), 7.81 (td, 1H, *J*_1_ = 1.6 Hz, *J*_2_ = 7.7 Hz), 7.97 (d, 1H, *J* = 8 Hz), 8.42 (m, 2H), 8.65 (d, 1H, *J* = 8 Hz). ^13^C NMR (100 MHz, CDCl_3_), δ, ppm: 14.35, 61.59, 73.22, 117.29, 118.93, 122.34, 127.14, 127.31, 128.34, 129.09, 133.63, 134.88, 140.47, 146.39, 147.11, 147.89, 159.15, 168.62. IR band, cm^−1^: 1686 (C=N), 963 (N-O), 1745 (C=O). Found, %: C 65.58, H 4.19, N 12.13. C_19_H_15_N_3_O_4_. Calculated, %: C 65.32, H 4.33, N 12.03.

*Diethyl 2-(((12-Oxoindolo[2,1-b]quinazolin-6(12H)-ylidene)amino)oxy)malonate* (**5b**). Yield 33%, colorless crystals, mp = 211 °C. ^1^H NMR (400 MHz, CDCl_3_), d, ppm: 1.34 (t, 6H, *J* = 7 Hz, CH_3_), 4.31 (q, 4H, *J* = 7 Hz, CH_2_CH_3_), 5.17 (s, 1H, =N-O-CH), 7.42 (td, 1H, *J*_1_ = 1 Hz, *J*_2_ = 7.7 Hz), 7.58-7.67 (m, 2H), 7.82 (td, 1H, *J*_1_ = 1.6 Hz, *J*_2_ = 6 Hz), 7.98 (d, 1H, *J* = 8 Hz), 8.44 (m, 2H), 8.68 (d, 1H, *J* = 8 Hz). ^13^C NMR (100 MHz, CDCl_3_), δ, ppm: 14.35, 61.58, 73.20, 117.29, 118.93, 122.33, 127.13, 127.31, 128.33, 129.05, 129.09, 133.62, 134.87, 140.47, 146.39, 147.11, 147.89, 159.14, 168.61. IR band, cm^−1^: 1687 (C=N), 964 (N-O), 1746 (C=O). Found, %: C 62.97, H 4.32, N 10.18. C_22_H_19_N_3_O_6_. Calculated, %: C 62.70, H 4.54, N 9.97.

#### 3.1.5. General Procedure for Synthesis of Compounds **5c**,**d**

TRYP-Ox (0.5 mmol) was dissolved at 0 °C in 6 mL of pyridine, chloroformate (0.5 mmol) was added, the mixture was stirred for 5 min, and precipitated in water. The precipitate that formed was filtered off, and the final compounds **5c** and **5d** were recrystallized from methanol.

*6-(((Methoxycarbonyl)oxy)imino)indolo[2,1-b]quinazolin-12(6H)-one* (**5c**). Yield 96%, mp = 197 °C. ^1^H NMR (400 Hz, acetone*-d*_6_), d, ppm: 3.77 (s, (3·0.67)H, CH_3_), 7.55 (td, 1H, *J*_1_ = 2.6 Hz, *J*_2_ = 7.8 Hz), 7.72 (td, 1H, *J*_1_ = 2 Hz, *J*_2_ = 7.8 Hz), 7.83 (td, 1H, *J*_1_ = 1.6 Hz, *J*_2_ = 8 Hz), 7.89–7.97 (m, 2H), 8.39 (dd, 2H, *J*_1_ = 1.6 Hz, *J*_2_ = 8 Hz), 8.67 (d, 1H, *J* = 8 Hz); the spectrum shows signals of the second geometric isomer at 4.07 (s, (3·0.33)H, CH_3_). IR band, cm^−1^: 1649 (C=N), 922 (N-O), 1749 (C=O); 1879 (-COOCH_3_). Found, %: C 63.63, H 3.35, N 12.92 C_17_H_11_N_3_O_4_. Calculated, %: C 63.55, H 3.45, N 13.08. LC/MS (ESI^+^)—*m*/*z*: 322.0820 experimental ([C_17_H_11_N_3_O_4_ + H]^+^ = 322.0822 theor.); *m*/*z*: 344.0642 exp. ([C_17_H_11_N_3_O_4_ + Na]^+^ = 344.0642 theor.); exit time 72–120 s.

*6-((((Prop-2-yn-1-yloxy)carbonyl)oxy)imino)indolo[2,1-b]quinazolin-12(6H)-one* (**5d**). Yield 38%, mp 210 °C. ^1^H NMR (400 Hz, CDCl_3_), d, ppm: 2.64 (t, 1H, *J* = 2.4 Hz, C≡CH), 5.00 (d, 2H, *J* = 2.4 Hz, CH_2_-C≡CH), 7.44 (td, 1H, *J*_1_ = 1.2 Hz, *J*_2_ = 7.8 Hz), 7.62 (td, 1H, *J*_1_ = 1.2 Hz, *J*_2_ = 7.8 Hz), 7.70 (td, 1H, *J*_1_ = 1.5 Hz, *J*_2_ = 7.9 Hz), 7.83 (td, 1H, *J*_1_ = 1.4 Hz, *J*_2_ = 7.9 Hz), 7.98 (d, 1H, *J* = 7 Hz), 8.39–8.44 (m, 2H), 8.65 (d, 1H, *J* = 8 Hz). IR band, cm^−1^: 1690 (C=N), 970 (N-O), 1791 (C=O). Found, %: C 66.37, H 3.12, N 12.36. C_19_H_11_N_3_O_4_. Calculated, %: C 66.09, H 3.21, N 12.17. LC/MS (ESI^+^)—*m*/*z*: 346.0820 experimental ([C_19_H_11_N_3_O_4_ + H]^+^ = 346.0822 theor.); *m*/*z*: 368.0642 exp. ([C_19_H_11_N_3_O_4_ + Na]^+^ = 368.0642 theor.); exit time 72–120 s.

#### 3.1.6. Preparation of 6-(((Benzo[*d*][1,3]dioxol-5-carbonyl)oxy)imino)indolo[2,1-*b*]quinazolin-12(6*H*)-one (**5e**)

Piperonyl chloride (**4c**) (0.0923 g, 0.5 mmol) was added to TRYP-Ox (0.132 g, 0.5 mmol) dissolved at 0 °C in 6 mL of pyridine, and the mixture was stirred for 60 min and precipitated in water. The resulting precipitate was dissolved in boiling methylene chloride and cooled to −40 °C. The precipitate formed from the solution was filtered on a chilled filter and washed with chilled methylene chloride. Yield 41%, mp 206–208 °C. ^1^H NMR (400 Hz, CDCl_3_), d, ppm: 6.15 (s, 2H, CH_2_), 6.98 (d, 1H, *J* = 8 Hz), 7.47 (td, 1H, *J*_1_ = 1.2 Hz, *J*_2_ = 7.6 Hz), 7.61–7.66 (m, 2H), 7.71 (td, 1H, *J*_1_ = 1.2 Hz, *J*_2_ = 8 Hz), 7.83-7.87 (m, 2H), 8.00 (d, 1H, *J* = 8 Hz), 8.35 (d, 1H, *J* = 8 Hz), 8.44 (dd, 1H, *J* = 1.6 Hz, *J* = 7.6 Hz), 8.71 (d, 1H, *J* = 8 Hz). IR band, cm^−1^: 1686 (C=N), 967 (N-O), 1754 (C=O); 1855 (-COOC-) Found, %: C 67.33, H 3.04, N 10.50. C_23_H_13_N_3_O_5_ Calculated, %: C 67.15, H 3.19, N 10.21. LC/MS (ESI^+^)—*m*/*z*: 412.0932 experimental ([C_23_H_13_N_3_O_5_ + H]^+^ = 412.0928 theor.); *m*/*z*: 434.0751 exp. ([C_23_H_13_N_3_O_5_ + Na]^+^ = 434.0747 theor.); exit time 72–120 s.

### 3.2. Biological Analysis

#### 3.2.1. Kinase K_d_ Determination

Compounds were submitted for dissociation constant (K_d_) determination using KINOMEscan (Eurofins Pharma Discovery, San Diego, CA, USA), as described previously [58]. Briefly, kinases were produced and displayed on T7 phages or expressed in HEK-293 cells. Binding reactions were performed at room temperature for 1 h, and the fraction of kinase not bound to the test compound was determined by capture with an immobilized affinity ligand and quantified by quantitative polymerase chain reaction. Primary screening at fixed concentrations of compounds was performed in duplicate. To determine dissociation constant K_d_, a 12-point half-log dilution series (a maximum concentration of 33 μM) was used. Assays were performed in duplicate, and their average mean value is displayed.

#### 3.2.2. Cell Culture

All cells were cultured at 37 °C in a humidified atmosphere containing 5% CO_2_. Human THP-1Blue monocytic cells obtained from InvivoGen (San Diego, CA, USA) were cultured in RPMI 1640 medium (Mediatech Inc., Herndon, VA, USA) supplemented with 10% (*v*/*v*) fetal bovine serum (FBS), 4.5 g/L glucose, 100 μg/mL streptomycin, 100 U/mL penicillin, 100 μg/mL phleomycin (Zeocin), and 10 μg/mL blasticidin S. Human monocyte-macrophage MonoMac-6 cells (Deutsche Sammlung von Mikroorganismen und Zellkulturen GmbH, Braunschweig, Germany) were grown in RPMI 1640 medium supplemented with 10% (*v*/*v*) FBS, 10 μg/mL bovine insulin, 100 μg/mL streptomycin, and 100 U/mL penicillin.

#### 3.2.3. Analysis of AP-1/NF-κB Activation

Activation of AP-1/NF-κB was measured using an alkaline phosphatase reporter gene assay in THP1-Blue cells. Human monocytic THP-1Blue cells were stably transfected with a secreted embryonic alkaline phosphatase gene that was under the control of a promoter inducible by AP-1/NF-κB. THP-1Blue cells (2 × 10^5^ cells/well) were pretreated with the test compound or DMSO (1% final concentration) for 30 min, followed by addition of 250 ng/mL LPS (from Escherichia coli K-235; Sigma Chemical Co., St. Louis, MO, USA) for 24 h, and alkaline phosphatase activity was measured in cell supernatants using QUANTI-Blue mix (InvivoGen) with absorbance at 655 nm and compared with positive control samples (LPS). The concentrations of compound that caused 50% inhibition of the AP-1/NF-κB reporter activity (IC50) were calculated.

#### 3.2.4. Cytotoxicity Assay

Cytotoxicity was analyzed with a CellTiter-Glo Luminescent Cell Viability Assay Kit from Promega (Madison, WI, USA), according to the manufacturer’s protocol. Cells were treated with the compound under investigation and cultivated for 24 h. After treatment, the cells were allowed to equilibrate to room temperature for 30 min, substrate was added, and the luminescence measured using a Fluoroscan Ascent FL (Thermo Fisher Scientific, Waltham, MA, USA). The cell IC_50_ values were calculated by plotting the percentage inhibition against the logarithm of inhibitor concentration (at least five points).

#### 3.2.5. Analysis of *c*-Jun Phosphorylation

MonoMac-6 cell lysates were prepared using Cell Lysis Buffer (Cell Signaling Technology, Danvers, MA, USA). The phospho-*c*-Jun content was measured with ELISA using the PathScan Phospho-*c*-Jun (Ser63) Sandwich ELISA Kit II (Cell Signaling Technology, USA), and the data are shown as the ratio of phospho-*c*-Jun vs. total *c*-Jun, which was measured using the FastScanTM Total *c*-Jun ELISA kit (Cell Signaling Technology).

### 3.3. Molecular Docking

The geometry of JNK3 was obtained by downloading its crystal structure from the Protein Data Bank (PDB entry code 1PMV) into Molegro 6.0 software (Molegro ApS, Aarhus, Denmark). All of the solvent molecules were removed, and the search space was chosen to be a sphere centered on the co-crystallized ligand present in the corresponding PDB structure. The radius of the sphere was 10 Å, which completely encompassed the co-crystallized ligand and the JNK3 binding site. Side chains of all JNK amino acid residues within the corresponding sphere were regarded as flexible during docking. The number of such residues was 39. The flexible residues were treated with the default settings of the “Setup Sidechain Flexibility” tool in Molegro, and a softening parameter of 0.7 was applied during flexible docking, according to the standard protocol using the Molegro Virtual Docker 6.0 (MVD). Before docking, structures of compounds were pre-optimized using HyperChem 7 software (HyperCube, Gainesville, FL, USA) with the MM+ force field, and saved in the Tripos MOL2 format (Tripos, St. Louis, MO, USA). The ligand structures were imported into MVD. The options “Create explicit hydrogens”, “Assign charges (calculated by MVD)”, and “Detect flexible torsions in ligands” were enabled during importing. Appropriate protonation states of the ligands were also automatically generated at this step. Each ligand was subjected to 30 docking runs with respect to the JNK3 structure using MVD software. The docking pose with the lowest MolDock docking score [69] was selected for each ligand and analyzed using the built-in tools of MVD.

### 3.4. DFT Calculations

ORCA 5.0 computational chemistry software [70] was used for density functional theory (DFT) calculations of compound **4d**. Geometry optimizations were performed using the M06-2X functional [65] with the 6-311++G(2d,2p) basis set. Normal vibration analysis was performed for the optimized geometries to establish the nature of the stationary points (i.e., minimum or transition state). Transition state (TS) geometry was obtained using the climbing image nudged elastic band (CI-NEB) approach [71] with the PBEh-3c composite method [72] followed by TS optimization at the M06-2X/6-311++G(2d,2p) level of theory. During CI-NEB calculations, eight intermediate images on the isomerization path were used. All calculations were performed using the conductor-like polarizable continuum solvation model (CPCM) [73] with DMSO as the solvent.

## 4. Conclusions

Based on a tryptanthrin scaffold, we designed and synthesized a series of substituted TRYP-Ox derivatives in an effort to obtain novel JNK3-selective inhibitors. As determined in our JNK1-3 binding assays, we were successful in identifying at least two highly selective JNK3 inhibitors. These compounds inhibited LPS-induced NF-κB/AP-1 transcription activity in THP-1Blue cells and IL-6 production in MonoMac-6 monocytic cells in the low micromolar range. In addition, the JNK3-selective inhibitors **4d** and **4e** decreased LPS-induced *c*-Jun phosphorylation in MonoMac-6 cells, directly confirming JNK inhibition. Molecular docking confirmed the binding interaction of the active compounds in the JNK3 catalytic site. These compounds may be useful for investigating the role of JNK3 in various models of pathologies such as Alzheimer’s disease, Parkinson’s disease, and cancer.

## Figures and Tables

**Table 1:** Chemical structures of starting isatins and isatoic anhydrides.

** 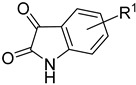 **				** 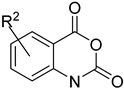 **	
**Compd.**	**R^1^**	**Compd.**	**R^1^**	**Compd.**	**R^2^**
**1a**	H	**1g**	4-CH_3_	**2a**	H
**1b**	5-NO_2_	**1h**	6-CH_3_	**2b**	6-Br
**1c**	5-Br	**1i**	5-CH_3_	**2c**	6-CF_3_
**1d**	5-Et	**1j**	6-OCH_3_	**2d**	7-Br
**1e**	5-OCH_3_	**1k**	6-Br	**2e**	7-f
**1f**	5-C_6_H_5_	**1l**	4-Br	**2f**	7-NO_2_
				**2g**	6-NO_2_

**Table 2:** Chemical structures of synthesized oxime derivatives and JNK binding affinity of the compounds.

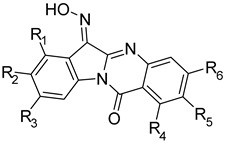											
**Compd.**	**R_1_**	**R_2_**	**R_3_**	**R_4_**	**R_5_**	**R_6_**	**JNK1**	**JNK2**	**JNK3**	**Selectivity Index**	
							**K_d_ (µM)**			**JNK1/JNK3**	**JNK2/JNK3**
TRYP-Ox	H	H	H	H	H	H	0.15 ± 0.08	1.0 ± 0.14	0.28 ± 0.21	0.15	3.6
**4a**	H	NO_2_	H	H	H	H	N.B.	N.B.	0.86 ± 0.042	>40	>40
**4b**	H	Br	H	H	H	H	3.2 ± 0.21	8.5 ± 3.2	0.41 ± 0.07	7.8	20.7
**4c**	H	Et	H	H	H	H	2.6 ± 0.4	1.3 ± 0.01	0.49 ± 0.09	5.3	2.7
**4d**	H	OMe	H	H	H	H	N.B.	N.B.	0.34 ± 0.03	>100	>100
**4e**	H	Ph	H	H	H	H	N.B.	N.B.	0.34 ± 0.04	>100	>100
**4f**	H	NO_2_	H	H	Br	H	N.B.	N.B.	N.B.		
**4g**	Me	H	H	H	H	H	6.2 ± 1.8	14.0 ± 0.7	0.40 ± 0.04	15.5	35
**4h**	H	H	Me	H	H	H	0.67 ± 0.35	0.57 ± 0.12	0.23 ± 0.04	2.9	2.5
**4i**	H	H	H	H	CF_3_	H	N.B.	N.B.	11.0 ± 1.4	>3	>3
**4j**	H	H	H	H	Br	H	4.1 ± 0.6	7.4 ± 0.6	1.9 ± 0.4	2.2	3.9
**4k**	H	H	H	H	H	Br	N.B.	N.B.	5.7 ± 1.8	>6	>6
**4l**	H	H	H	H	H	F	1.1 ± 0.1	0.72 ± 0.22	0.43 ± 0.04	2.6	1.7
**4m**	H	H	H	H	H	NO_2_	N.B.	N.B.	6.1 ± 0.4	>5.6	>5.6
**4n**	H	H	H	H	NO_2_	H	N.B.	N.B.	8.7 ± 1.0	>3.9	>3.9
**4o**	H	Me	H	H	Me	H	3.7 ± 0.5	6.9 ± 0.2	0.87 ± 0.16	4.3	7.9
**4p**	Me	H	H	Me	H	H	N.B.	N.B.	N.B.		
**4q**	H	OMe	H	H	OMe	H	N.B.	N.B.	N.B.		
**4r**	H	H	OMe	H	H	OMe	4.6 ± 0.6	5.8 ± 1.6	3.2 ± 1.1	1.4	1.8
**4s**	H	H	Br	H	H	Br	N.B.	N.B.	N.B.		
**4t**	H	Br	H	H	Br	H	N.B.	N.B.	N.B.		
**4u**	Br	H	H	Br	H	H	N.B.	N.B.	N.B.		
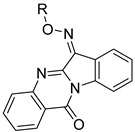											
**Compd.**	**R**						**JNK1**	**JNK2**	**JNK3**	**Selectivity Index**	
							**K_d_ (µM)**			**JNK1/JNK3**	**JNK2/JNK3**
**5a**	-CH_2_COOEt						N.B.	N.B.	N.B.		
**5b**	-CH(COOEt)_2_						16.5 ± 0.7	N.B.	N.B.		
**5c**	-COOCH_3_						1.7 ± 0.4	2.8 ± 0.5	0.70 ± 0.12	2.4	4.0
**5d**	-COOCH_2_-C≡CH						0.95 ± 0.03	1.6 ± 0.1	0.32 ± 0.06	3.0	5.0
**5e**	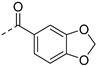						0.47 ± 0.08	1.5 ± 0.6	0.27 ± 0.01	1.7	0.9

N.B., no binding affinity at concentrations < 30 µM.

**Table 3:** Summary of compound cytotoxic activity and their inhibitory effects on LPS-induced NF-κB/AP-1 transcriptional activity in THP-1Blue cells and IL-6 production in MonoMac-6 cells.

Compd.	AP Production in THP–1Blue	Cytotoxicity in THP–1Blue	IL–6 Production in MonoMac–6	Cytotoxicity in MonoMac–6
	IC_50_, μM			
TRYP–Ox	3.8 ± 1.1	N.T.	3.2 ± 1.2	N.T.
**4a**	n.d.	8.4 ± 0.9	n.d.	4.1 ± 0.7
**4b**	2.1 ± 0.7	N.T.	15.5 ± 3.8	N.T.
**4c**	0.7 ± 0.2	N.T.	n.d.	13.3 ± 1.3
**4d**	0.8 ± 0.3	N.T.	0.7 ± 0.1	N.T.
**4e**	1.6 ± 0.1	N.T.	0.17 ± 0.02	N.T.
**4f**	n.d.	3.8 ± 0.9	n.d.	2.7 ± 0.6
**4g**	N.A.	N.T.	25.2 ± 5.3	N.T.
**4h**	0.9 ± 0.2	N.T.	0.3 ± 0.1	N.T.
**4i**	n.d.	5.6 ± 1.2	n.d.	5.7 ± 1.2
**4j**	n.d.	12.5 ± 2.3	n.d.	8.2 ± 1.6
**4k**	n.d.	24.1 ± 2.7	0.41 ± 0.04	N.T.
**4l**	5.0 ± 0.1	N.T.	0.61 ± 0.04	N.T.
**4m**	8.3 ± 0.2	N.T.	1.9 ± 0.3	N.T.
**4n**	n.d.	39.3 ± 3.1	9.6 ± 1.3	N.T.
**4o**	8.1 ± 0.1	N.T.	0.8 ± 0.1	N.T.
**4p**	N.A.	N.T.	N.A.	N.T.
**4q**	4.6 ± 0.3	N.T.	1.3 ± 0.4	N.T.
**4r**	n.d.	8.3 ± 1.3	n.d.	4.3 ± 0.9
**4s**	n.d.	49.1 ± 8.3	n.d.	16.9 ± 3.4
**4t**	n.d.	6.6 ± 0.3	n.d.	9.2 ± 2.1
**4u**	N.A.	N.T.	N.A.	N.T.
**5a**	N.A.	N.T.	N.A.	N.T.
**5b**	N.A.	N.T.	N.A.	N.T.
**5c**	8.1 ± 2.2	N.T.	12.4 ± 1.3	N.T.
**5d**	4.4 ± 1.6	N.T.	7.0 ± 0.9	N.T.
**5e**	3.5 ± 1.2	N.T.	n.d.	27.3 ± 1.4

N.T., no cytotoxicity was found at concentrations up to 50 µM; n.d., not determined whether a compound was cytotoxic in the cell line; N.A.: No activity was observed at the highest concentration tested (50 μM).

**Table 4:** MolDock docking scores (DS) for the best docking poses of compounds into the binding site of JNK3 (PDB: 1PMV).

Compound	DS
TRYP-Ox (*Z*)	−62.2
TRYP-Ox (*E*)	−34.9
**4a** (*Z*)	−71.12
**4a** (*E*)	−70.17
**4b** (*Z*)	−73.32
**4b** (*E*)	−66.26
**4d** (*E*)	−84.91
**4d** (*Z*)	*
**4f** (*Z*)	−51.87
**4f** (*E*)	−27.04
**4j** (*E*)	−70.95
**4j** (*Z*)	−63.44
**4k** (*Z*)	−71.36
**4k** (*E*)	*
**4m** (*Z*)	−69.18
**4m** (*E*)	−44.17
**4n** (*Z*)	−65.97
**4n** (*E*)	−38.77
**4q** (*Z*)	−34.07
**4q** (*E*)	−23.00
**4s** (*E*)	−49.47
**4s** (*Z*)	−21.43
**4t** (*E*)	−29.88
**4t** (*Z*)	−16.65
**4u** (*E*)	−45.07
**4u** (*Z*)	−21.39

* No docking pose with a negative DS value was found. Isomer pairs are highlighted.

## Data Availability

Data are contained within the article.

## Sample Availability

Samples of synthesized compounds are available from the authors.

## References

[1] Kyriakis J.M., Banerjee P., Nikolakaki E., Dai T., Rubie E.A., Ahmad M.F., Avruch J., Woodgett J.R. (1994). The stress-activated protein kinase subfamily of c-Jun kinases. Nature.

[2] Abdelrahman K.S., Hassan H.A., Abdel-Aziz S.A., Marzouk A.A., Narumi A., Konno H., Abdel-Aziz M. (2021). JNK signaling as a target for anticancer therapy. Pharmacol. Rep..

[3] Johnson G.L., Lapadat R. (2002). Mitogen-activated protein kinase pathways mediated by ERK, JNK, and p38 protein kinases. Science.

[4] Lowes V.L., Ip N.Y., Wong Y.H. (2002). Integration of signals from receptor tyrosine kinases and G protein-coupled receptors. Neurosignals.

[5] Hammouda M.B., Ford A.E., Liu Y., Zhang J.Y. (2020). The JNK Signaling Pathway in Inflammatory Skin Disorders and Cancer. Cells.

[6] Ip Y.T., Davis R.J. (1998). Signal transduction by the c-Jun N-terminal kinase (JNK)—From inflammation to development. Curr. Opin. Cell Biol..

[7] Javadov S., Jang S., Agostini B. (2014). Crosstalk between mitogen-activated protein kinases and mitochondria in cardiac diseases: Therapeutic perspectives. Pharmacol. Ther..

[8] Nijboer C.H., van der Kooij M.A., van Bel F., Ohl F., Heijnen C.J., Kavelaars A. (2010). Inhibition of the JNK/AP-1 pathway reduces neuronal death and improves behavioral outcome after neonatal hypoxic-ischemic brain injury. Brain Behav. Immun..

[9] Geng C., Wei J., Wu C. (2019). Mammalian STE20-like kinase 1 knockdown attenuates tnfalpha-mediated neurodegenerative disease by repressing the JNK pathway and mitochondrial stress. Neurochem. Res..

[10] Johnson G.L., Nakamura K. (2007). The c-jun kinase/stress-activated pathway: Regulation, function and role in human disease. Biochim. Biophys. Acta.

[11] Waetzig V., Herdegen T. (2005). Context-specific inhibition of JNKs: Overcoming the dilemma of protection and damage. Trends Pharmacol. Sci..

[12] Guma M., Ronacher L.M., Firestein G.S., Karin M., Corr M. (2011). JNK-1 deficiency limits macrophage-mediated antigen-induced arthritis. Arthritis Rheum..

[13] Bogoyevitch M.A., Boehm I., Oakley A., Ketterman A.J., Barr R.K. (2004). Targeting the JNK MAPK cascade for inhibition: Basic science and therapeutic potential. Biochim. Biophys. Acta.

[14] Zhang G.Y., Zhang Q.G. (2005). Agents targeting c-Jun N-terminal kinase pathway as potential neuroprotectants. Expert Opin. Investig. Drugs.

[15] Ge H.X., Zou F.M., Li Y., Liu A.M., Tu M. (2017). JNK pathway in osteoarthritis: Pathological and therapeutic aspects. J. Recept. Sig. Transd..

[16] Solinas G., Becattini B. (2017). JNK at the crossroad of obesity, insulin resistance, and cell stress response. Mol. Metab..

[17] Kumar A., Singh U.K., Kini S.G., Garg V., Agrawal S., Tomar P.K., Pathak P., Chaudhary A., Gupta P., Malik A. (2015). JNK pathway signaling: A novel and smarter therapeutic targets for various biological diseases. Future Med. Chem..

[18] Wu Q.H., Wu W.D., Jacevic V., Franca T.C.C., Wang X., Kuca K. (2020). Selective inhibitors for JNK signalling: A potential targeted therapy in cancer. J. Enzym. Inhib. Med. Chem..

[19] Plotnikov M.B., Aliev O.I., Shamanaev A.Y., Sidekhmenova A.V., Anishchenko A.M., Fomina T.I., Rydchenko V.S., Khlebnikov A.I., Anfinogenova Y.J., Schepetkin I.A. (2020). Antihypertensive activity of a new c-Jun N-terminal kinase inhibitor in spontaneously hypertensive rats. Hypertens. Res..

[20] Plotnikov M.B., Chernysheva G.A., Aliev O.I., Smol’iakova V.I., Fomina T.I., Osipenko A.N., Rydchenko V.S., Anfinogenova Y.J., Khlebnikov A.I., Schepetkin I.A. (2019). Protective effects of a new c-Jun N-terminal kinase inhibitor in the model of global cerebral ischemia in rats. Molecules.

[21] Plotnikov M.B., Chernysheva G.A., Smolyakova V.I., Aliev O.I., Trofimova E.S., Sherstoboev E.Y., Osipenko A.N., Khlebnikov A.I., Anfinogenova Y.J., Schepetkin I.A. (2020). Neuroprotective effects of a novel inhibitor of c-Jun N-terminal kinase in the rat model of transient focal cerebral ischemia. Cells.

[22] Cho H., Hah J.M. (2021). A Perspective on the development of c-Jun N-terminal kinase inhibitors as therapeutics for Alzheimer’s Disease: Investigating Structure through Docking Studies. Biomedicines.

[23] Gehringer M., Muth F., Koch P., Laufer S.A. (2015). c-Jun *N*-terminal kinase inhibitors: A patent review (2010–2014). Expert Opin. Ther. Pat..

[24] Rehfeldt S.C.H., Majolo F., Goettert M.I., Laufer S. (2020). c-Jun N-terminal kinase inhibitors as potential leads for new therapeutics for Alzheimer’s Diseases. Int. J. Mol. Sci..

[25] Ha J., Kang E., Seo J., Cho S. (2019). Phosphorylation Dynamics of JNK signaling: Effects of dual-specificity phosphatases (DUSPs) on the JNK pathway. Int. J. Mol. Sci..

[26] Bode A.M., Dong Z. (2007). The functional contrariety of JNK. Mol. Carcinog..

[27] Gupta S., Barrett T., Whitmarsh A.J., Cavanagh J., Sluss H.K., Derijard B., Davis R.J. (1996). Selective interaction of JNK protein kinase isoforms with transcription factors. EMBO J..

[28] Abdelli S., Bonny C. (2012). JNK3 Maintains expression of the insulin receptor substrate 2 (IRS2) in insulin-secreting cells: Functional consequences for insulin signaling. PLoS ONE.

[29] Zhou Y., Zhang P.X., Zheng X.Y., Ye C.T., Li M.Y., Bian P.Y., Fan C., Zhang Y. (2021). miR-155 regulates pro- and anti-inflammatory cytokine expression in human monocytes during chronic hepatitis C virus infection. Ann. Transl. Med..

[30] Gorogh T., Beress L., Quabius E.S., Ambrosch P., Hoffmann M. (2013). Head and neck cancer cells and xenografts are very sensitive to palytoxin: Decrease of c-jun n-terminale kinase-3 expression enhances palytoxin toxicity. Mol. Cancer.

[31] Lapouge G., Millon R., Muller D., Abecassis J., Eber M., Bergerat J.P., Klein-Soyer C. (2005). Cisplatin-induced genes as potential markers for thyroid cancer. Cell. Mol. Life Sci..

[32] Pi X., Wu Y., Ferguson J.E., Portbury A.L., Patterson C. (2009). SDF-1α stimulates JNK3 activity via eNOS-dependent nitrosylation of MKP7 to enhance endothelial migration. Proc. Natl. Acad. Sci. USA.

[33] Lattin J.E., Greenwood K.P., Daly N.L., Kelly G., Zidar D.A., Clark R.J., Thomas W.G., Kellie S., Craik D.J., Hume D.A. (2009). Beta-arrestin 2 is required for complement C1q expression in macrophages and constrains factor-independent survival. Mol. Immunol..

[34] Ebelt N.D., Cantrell M.A., van den Berg C.L. (2013). c-Jun N-terminal kinases mediate a wide range of targets in the metastatic cascade. Genes Cancer.

[35] Yang X., Xiang X., Xia M., Su J., Wu Y., Shen L., Xu Y., Sun L. (2015). Inhibition of JNK3 promotes apoptosis induced by BH3 mimetic S1 in chemoresistant human ovarian cancer cells. Anat. Rec..

[36] Yan F., Wang X.M., Liu Z.C., Pan C., Yuan S.B., Ma Q.M. (2010). JNK1, JNK2, and JNK3 are involved in P-glycoprotein-mediated multidrug resistance of hepatocellular carcinoma cells. Hepatobiliary Pancreat. Dis. Int..

[37] Butterfield L., Zentrich E., Beekman A., Heasley L.E. (1999). Stress- and cell type-dependent regulation of transfected c-Jun N-terminal kinase and mitogen-activated protein kinase kinase isoforms. Biochem. J..

[38] Sun R., Xiang T.X., Tang J., Peng W.Y., Luo J., Li L.L., Qiu Z., Tan Y.Q., Ye L., Zhang M. (2020). 19q13 KRAB zinc-finger protein ZNF471 activates MAPK10/JNK3 signaling but is frequently silenced by promoter CpG methylation in esophageal cancer. Theranostics.

[39] Finch A., Davis W., Carter W.G., Saklatvala J. (2001). Analysis of mitogen-activated protein kinase pathways used by interleukin 1 in tissues in vivo: Activation of hepatic c-Jun N-terminal kinases 1 and 2, and mitogen-activated protein kinase kinases 4 and 7. Biochem. J..

[40] Gorogh T., Berwig J., Scola N., Lippert B.M. (2004). Differential regulation of MAPK (JNK 3) gene expression in human head and neck squamous cell carcinomas. Onkologie.

[41] Zang Y.C., Zhu J., Li Q., Tu J., Li X.Q., Hu R.K., Yang D.R. (2020). miR-137-3p modulates the progression of prostate cancer by regulating the JNK3/EZH2 axis. OncoTargets Ther..

[42] Wu X., Chen S., Lu C. (2020). Amyloid precursor protein promotes the migration and invasion of breast cancer cells by regulating the MAPK signaling pathway. Int. J. Mol. Med..

[43] Wang D.R., Pei P., Shea F.F., Bissonnette C., Nieto K., Din C., Liu Y.Y., Schwendeman S.P., Lin Y.X., Richard S. (2022). Fenretinide combines perturbation of signaling kinases, cell-extracellular matrix interactions and matrix metalloproteinase activation to inhibit invasion in oral squamous cell carcinoma cells. Carcinogenesis.

[44] Rehfeldt S.C.H., Laufer S., Goettert M.I. (2021). A highly selective in vitro JNK3 inhibitor, FMU200, restores mitochondrial membrane potential and reduces oxidative stress and apoptosis in SH-SY5Y cells. Int. J. Mol. Sci..

[45] Li Z.T., Zhu G.W., Liu X.A., Gao T.F., Fang F., Dou X.D., Li Y.Y., Zheng R.Q., Jin H.W., Zhang L.R. (2023). The structure-based optimization of 3-substituted indolin-2-one derivatives as potent and isoform-selective c-Jun N-terminal kinase 3 (JNK3) inhibitors and biological evaluation. Eur. J. Med. Chem..

[46] Shuai W., Bu F.Q., Zhu Y.M., Wu Y.Y., Xiao H., Pan X.L., Zhang J.F., Sun Q., Wang G., Ouyang L. (2023). Discovery of novel indazole chemotypes as isoform-selective JNK3 inhibitors for the treatment of Parkinson’s Disease. J. Med. Chem..

[47] Jun J., Baek J., Kang D., Moon H., Kim H., Cho H., Hah J.M. (2023). Novel 1,4,5,6-tetrahydrocyclopenta[d]imidazole-5-carboxamide-based JNK3 inhibitors: Design, synthesis, molecular docking, and therapeutic potential in neurodegenerative diseases. Eur. J. Med. Chem..

[48] Kaur R., Manjal S.K., Rawal R.K., Kumar K. (2017). Recent synthetic and medicinal perspectives of tryptanthrin. Bioorganic Med. Chem..

[49] Schepetkin I.A., Kovrizhina A.R., Stankevich K.S., Khlebnikov A.I., Kirpotina L.N., Quinn M.T., Cook M.J. (2022). Design, synthesis and biological evaluation of novel O-substituted tryptanthrin oxime derivatives as c-Jun N-terminal kinase inhibitors. Front. Pharmacol..

[50] Schepetkin I.A., Khlebnikov A.I., Potapov A.S., Kovrizhina A.R., Matveevskaya V.V., Belyanin M.L., Atochin D.N., Zanoza S.O., Gaidarzhy N.M., Lyakhov S.A. (2019). Synthesis, biological evaluation, and molecular modeling of 11H-indeno[1,2-b]quinoxalin-11-one derivatives and tryptanthrin-6-oxime as c-Jun N-terminal kinase inhibitors. Eur. J. Med. Chem..

[51] Kirpotina L.N., Schepetkin I.A., Hammaker D., Kuhs A., Khlebnikov A.I., Quinn M.T. (2020). Therapeutic effects of tryptanthrin and tryptanthrin-6-oxime in models of rheumatoid arthritis. Front. Pharmacol..

[52] Nie Z.L., Xia X.L., Zhao Y., Zhang S., Zhang Y.W., Wang J.H. (2021). JNK selective inhibitor, IQ-1S, protects the mice against lipopolysaccharides-induced sepsis. Bioorgan. Med. Chem..

[53] Schepetkin I.A., Kirpotina L.N., Khlebnikov A.I., Hanks T.S., Kochetkova I., Pascual D.W., Jutila M.A., Quinn M.T. (2012). Identification and characterization of a novel class of c-Jun N-terminal kinase inhibitors. Mol. Pharmacol..

[54] Liakhov S.A., Schepetkin I.A., Karpenko O.S., Duma H.I., Haidarzhy N.M., Kirpotina L.N., Kovrizhina A.R., Khlebnikov A.I., Bagryanskaya I.Y., Quinn M.T. (2021). Novel c-Jun N-Terminal Kinase (JNK) Inhibitors with an 11H-Indeno[1,2-b]quinoxalin-11-one Scaffold. Molecules.

[55] Jia F.C., Zhou Z.W., Xu C., Wu Y.D., Wu A.X. (2016). Divergent synthesis of quinazolin-4(3H)-ones and tryptanthrins enabled by a tert-butyl hydroperoxide/K_3_PO_4_-promoted oxidative cyclization of isatins at room temperature. Org. Lett..

[56] Tucker A.M., Grundt P. (2012). The chemistry of tryptanthrin and its derivatives. Arkivoc.

[57] Trofimov B.A., Schmidt E.Y. (2014). Reactions of acetylenes in superbasic media. Recent advances. Russ. Chem. Rev..

[58] Karaman M.W., Herrgard S., Treiber D.K., Gallant P., Atteridge C.E., Campbell B.T., Chan K.W., Ciceri P., Davis M.I., Edeen P.T. (2008). A quantitative analysis of kinase inhibitor selectivity. Nat. Biotechnol..

[59] Ansideri F., Dammann M., Boeckler F.M., Koch P. (2017). Fluorescence polarization-based competition binding assay for c-Jun N-terminal kinases 1 and 2. Anal. Biochem..

[60] Guha M., Mackman N. (2001). LPS induction of gene expression in human monocytes. Cell. Signal..

[61] Takeuchi O., Akira S. (2001). Toll-like receptors; their physiological role and signal transduction system. Int. Immunopharmacol..

[62] Dugave C., Demange L. (2003). Cis-trans isomerization of organic molecules and biomolecules: Implications and applications. Chem. Rev..

[63] Blanco F., Alkorta I., Elguero J. (2009). Barriers about double carbon-nitrogen bond in imine derivatives (aldimines, oximes, hydrazones, azines). Croat. Chem. Acta.

[64] Scapin G., Patel S.B., Lisnock J., Becker J.W., LoGrasso P.V. (2003). The structure of JNK3 in complex with small molecule inhibitors: Structural basis for potency and selectivity. Chem. Biol..

[65] Zhao Y., Truhlar D.G. (2008). The M06 suite of density functionals for main group thermochemistry, thermochemical kinetics, noncovalent interactions, excited states, and transition elements: Two new functionals and systematic testing of four M06-class functionals and 12 other functionals. Theor. Chem. Acc..

[66] Weiss K., Warren C.H., Wettermark G. (1971). Cis-trans isomerization about the carbon-nitrogen double bond. Structures of the isomers of N-benzylideneaniline. J. Am. Chem. Soc..

[67] Lakeev A.P., Frelikh G.A., Yanovskaya E.A., Kovrizhina A.R., Udut V.V. (2022). Quantification of a promising JNK inhibitor and nitrovasodilator IQ-1 and its major metabolite in rat plasma by LC-MS/MS. Bioanalysis.

[68] Krivogorsky B., Nelson A.C., Douglas K.A., Grundt P. (2013). Tryptanthrin derivatives as *Toxoplasma gondii* inhibitors-structure-activity-relationship of the 6-position. Bioorganic Med. Chem. Lett..

[69] Thomsen R., Christensen M.H. (2006). MolDock: A new technique for high-accuracy molecular docking. J. Med. Chem..

[70] Neese F., Wennmohs F., Becker U., Riplinger C. (2020). The ORCA quantum chemistry program package. J. Chem. Phys..

[71] Ásgeirsson V., Birgisson B.O., Bjornsson R., Becker U., Neese F., Riplinger C., Jónsson H. (2021). Nudged Elastic Band Method for Molecular Reactions Using Energy-Weighted Springs Combined with Eigenvector Following. J. Chem. Theory Comput..

[72] Grimme S., Brandenburg J.G., Bannwarth C., Hansen A. (2015). Consistent structures and interactions by density functional theory with small atomic orbital basis sets. J. Chem. Phys..

[73] Skyner R.E., McDonagh J.L., Groom C.R., van Mourik T., Mitchell J.B. (2015). A review of methods for the calculation of solution free energies and the modelling of systems in solution. Phys. Chem. Chem. Phys..

